# Host-specific platelet-activating factor acetylhydrolase selectively remodels diacylglycerophospholipids to control schistosome development

**DOI:** 10.1371/journal.ppat.1014207

**Published:** 2026-05-12

**Authors:** Johanna Ertl, Ulrich Fabien Prodjinotho, Zhigang Rao, Anisuzzaman, Julia Schluckebier, Youssef Hamway, Paula Baar, Haifu Zhao, Martin Haslbeck, Christoph G. Grevelding, Simone Haeberlein, Franco H. Falcone, Sabine Schulz, Andreas Koeberle, Clarissa Prazeres da Costa

**Affiliations:** 1 Institute for Medical Microbiology, Immunology and Hygiene, TUM School of Medicine and Health, Technical University of Munich (TUM), Munich, Germany; 2 Center for Global Health, TUM School of Medicine and Health, Technical University of Munich (TUM), Munich, Germany; 3 Michael Popp Institute and Center for Molecular Biosciences Innsbruck (CMBI), Innsbruck, Austria; 4 Department of Parasitology, Bangladesh Agricultural University, Mymensingh, Bangladesh; 5 German Centre for Infection Research (DZIF), Partner Site Munich, Munich, Germany; 6 Institute of Inorganic and Analytical Chemistry, Justus Liebig University Giessen, Giessen, Germany; 7 Center for Functional Protein Assemblies and Department of Bioscience, School of Natural Sciences, Technical University of Munich (TUM), Garching, Germany; 8 Institute of Parasitology, BFS, Justus Liebig University Giessen, Giessen, Germany; 9 Institute of Pharmaceutical Sciences and Excellence Field BioHealth, University of Graz, Graz, Austria; George Washington University, UNITED STATES OF AMERICA

## Abstract

**Background:**

Schistosomiasis, one of the most prevalent neglected tropical diseases, is treated exclusively with praziquantel, which targets only adult worms. High reinfection rates and the potential emergence of drug resistance emphasize the need for a deeper understanding of host-parasite developmental biology for innovative therapeutic targets. Most studies have focused on parasite-derived immunomodulators and proteolytic enzymes essential for host invasion, while the contribution of host-derived factors to parasite development has remained largely unexplored. Here, we identify and functionally characterize a host phospholipase that modulates parasite diacylglycerophospholipid metabolism and development.

**Methods:**

Large-scale proteomic screening identified host platelet-activating factor acetylhydrolase (PAFAH) as a candidate schistosomicidal factor. Using a mouse model of schistosomiasis, we produced recombinant mouse PAFAH (MsPAFAH) and performed *ex vivo* killing assays on juvenile stages and adult worms. Parasite morphology and reproductive organ integrity were assessed by confocal and scanning electron microscopy. Alterations in glycerophospholipid distribution were quantified by UPLC-MS/MS-based fatty acid profiling, followed by rescue experiments with free fatty acid supplementation.

**Results:**

MsPAFAH was upregulated in schistosome-infected mice and exhibited potent schistosomicidal activity against all life stages in the host. MsPAFAH treatment led to profound impairments in worm fecundity, pairing stability, reproductive organ integrity, and stem cell development. This activity was associated with substantial sex-dependent changes in ether-phospholipid composition and distribution within the schistosome. MsPAFAH specifically decreased the availability of phospholipid species containing namely eicosenoic (20:1) and docosatetraenoic acid (22:4), while increasing levels of respective hydrolysis (lyso) products carrying 20:1 or stearic acid (18:0), predominantly in males. Supplementation of metabolized fatty acids C20:1/eicosenoic acid and 22:4/adrenic acid rescued the viability of female worms, confirming the essential role of the metabolism of these diacylglycerophospholipids in schistosome survival.

**Conclusions:**

Our findings reveal a previously unrecognized host-derived regulator of schistosome development, highlighting phospholipid metabolism as a promising therapeutic axis for consideration in schistosomiasis interventions.

## Author summary

Schistosomiasis is one of the most widespread parasitic diseases worldwide, affecting millions of people, mostly in resource-limited regions. Treatment relies on a single drug, praziquantel, which works only against adult worms. This limitation leaves the earlier developmental stages unaffected and contributes to frequent reinfections. Most research to date has focused on parasite molecules that help the worms invade and manipulate the immune system. In contrast, very little is known about how host molecules influence parasite survival.

In our work, we identified a host enzyme, platelet-activating factor acetylhydrolase (PAFAH), that dramatically affects the survival of the schistosome parasite. We discovered that the mouse version of this enzyme, not the human one, is increased during infection and can kill worms at every life stage, including the young stages usually unaffected by treatment. We showed that this enzyme works by interfering with the parasite’s lipid metabolism, removing specific fatty acids that are essential for the worms’ development and reproduction. Remarkably, adding precursors of these fatty acids rescued parasite survival.

Our findings reveal a new way, whereby the host can influence parasite biology by reshaping its lipids. This opens a new avenue for developing treatments that could work at multiple stages of the infection.

## Introduction

Schistosomiasis, one of the 20 neglected tropical diseases (NTD), is caused by parasitic trematodes of the genus *Schistosoma* and is a significant global health challenge, affecting over 210 million people in tropical and subtropical regions worldwide [1]. Despite concerted efforts to control schistosomiasis, it continues to exert a considerable burden on affected populations, particularly in resource-limited settings in sub-Saharan Africa, where it is mostly endemic. However, the limitations of PZQ, including its ineffectiveness against early larval stages and the potential emergence of drug resistance, underscore the urgent need for alternative therapeutic approaches for the populations at risk.

Schistosomiasis affects tropical and subtropical areas, and an estimated 800 million, mostly children, are at risk [1]. The global burden of schistosomiasis is attributed to 3.31 million disability-adjusted life years (DALYs) per year [2]. Thousands of deaths occur each year [3], and additionally, several hundred million people are struggling with residual post-treatment morbidity. In the regions with typical transmission patterns, 60–80% of school-age children and 20–40% of adults can remain actively infected despite mass drug administration (MDA) campaigns [4]. Although autochthonous schistosomiasis in non-endemic regions, such as Europe, is unexpected, recent studies reported transmission in Southern Europe, indicating the probability of schistosomiasis also establishing in more moderate climate zones [5,6]. Given the vast use of a single drug only, there is concern about the emergence of drug resistance, which has been demonstrated experimentally and in a limited number of field samples [7,8]. Indeed, the WHO, in its 2030 roadmap to fight NTDs, has specifically outlined this concern to better understand the schistosome biology within the host and motivate the development of new drugs [9].

For over 30 years, in-depth investigations of mechanisms of host-parasite interaction and underlying immunopathologies *in vivo* have mainly relied on the *Schistosoma mansoni* mouse model [10]. In this model, however, only about 30% of penetrated cercariae mature into fecund adult worms as mice are not the definite host for *S. mansoni* and differences in host susceptibility have been observed [10–12]. We previously revealed that this loss of cercariae was not mediated by adaptive immune cells or by major factors of the complement system, like C3, C4, or C1q, or antibodies but discovered an unexpectedly strong schistomicidal effect of mouse serum on all developmental parasite stages *in vitro* [10]. From the penetration of cercariae, schistosomes continuously stay in contact and bathe in the mammalian blood. Thus, it is pertinent that soluble serum factors have an obvious role in parasite development and could contribute to the host specificity of this blood-dwelling fluke [10,11]. However, most investigations so far have targeted parasite immunomodulatory and proteolytic molecules and enzymes, critical in facilitating host invasion, nutrient uptake, hatching, immune system evasion, and host physiology modulation [11,13]. In contrast, only very few host-derived proteins and their interactions with parasites have been functionally characterized [11,14].

Among the prominent factors in host serum, enzymes play essential roles in regulating energy levels. By catalyzing the hydrolysis and modification of key molecules to provide nutrients, generating bioactive mediators, and influencing cell metabolism, host enzymes, e.g., human lipoxygenases and peroxiredoxins, are central to the survival of parasites [11,15–17]. As such, host enzymes are, in general, essential targets for regulating cell metabolism in various research areas [18]. In cancer research, targeting glucose and lipid metabolic enzymes has emerged as a promising strategy for developing antineoplastic drugs [19,20]. In helminths, especially for schistosomes, lipid metabolism plays a crucial role in the survival and pathogenesis of the worm, making it a relevant target for novel therapeutic strategies [11,21,22]. Schistosomes rely on lipid metabolism to acquire essential nutrients for their growth, development, and reproduction, e.g., for, egg production and maturation [23–25]. Essentially, lipids and phospholipids (PL) are important components of the schistosome membrane, contributing to its structure, integrity, functions, and host immune evasion [22,26]. Furthermore, schistosomes are unable to synthesize fatty acids and sterols *de novo* [25,26]. Hence, disruption of schistosome PL metabolism can impair nutrient uptake, ion transport, and communication with the host environment, ultimately leading to parasite death [15,25]. Thus, host phospholipases, a group of enzymes capable of hydrolyzing PL, are particularly interesting as they potentially represent anti-schistosomal molecules [11,23].

In this study, through multifaceted molecular approaches and proteomic analyses, we unmasked that the lipoprotein-associated phospholipase A_2_, platelet-activating factor-acetylhydrolase (PAFAH) of host origin, is one of the major serum factors, which critically influences the survival of mammalian host-dwelling stages of schistosomes. We demonstrated *ex vivo* that recombinant mouse, but not human, PAFAH, disrupts tegument integrity and essential parasite structures, interferes with lipid metabolism and homeostasis, and impairs key physiological processes crucial for parasite survival and development. These findings shed light on mechanisms underlying host preference and specificity of schistosomes and enable further research into the identification of the mode of action of PAFAH. This may guide the development of novel drugs, such as small molecules interfering specifically with the PL metabolism of the parasite.

## Materials and methods

### Ethics statements

The animal and mouse serum investigations were approved by the Bezirksregierung Oberbayern (license number AZ 55.2-1-54-2532-145-17). Investigations using human serum were approved by the local ethical committee of the Technical University of Munich (TUM) (Reference: 215/18S), and written formal consent was obtained from all individuals, included in the study.

### Animals, blood samples, and serum preparation

NMRI mice were purchased (Envigo, Germany) or bred in-house. Animals were maintained according to national and EU guidelines 86/809 under specific pathogen-free conditions at the Institute for Medical Microbiology, Immunology, and Hygiene (MIH) Animal facility. Animals (6–8 weeks of age) of both sexes were used. Sera from infected and non-infected mice were prepared from blood collected by venipuncture in non-medicated Falcon tubes. The blood was centrifuged for five minutes, and serum was collected separately or pooled when indicated and stored at -20°C until use.

Human serum was prepared from the blood of healthy female and male volunteers with no previous history of schistosomiasis. Fresh blood was clotted at room temperature for 30 min and centrifuged at 1,845 x g for 20 min. Serum was collected, pooled, and stored at -20°C until further use.

### NTS generation, culture, and advanced larval stage development

Newly Transformed Schistosomula (NTS) were generated as previously described [27,28]. For *in vitro* culture, approximately 100 NTS in 150 µl HybridoMed (HM) culture medium (HybridoMed Diff 1000 (Biochrom GmbH, Germany) supplemented with 200 U/mL Penicillin, 200 µg/mL Streptomycin (Sigma-Aldrich) were plated in 96-well flat-bottom tissue culture plate and incubated at 37 °C in 5% CO_2_ atmosphere to rest for 48 hours. For long-term culture and advance stage development, 100 NTS were resuspended in HM supplemented with 20% human serum (HM + HSe) and cultured at 37   °C, 5% CO_2_ atmosphere for 25–30 days post-transformation (p.t.) [27]. To guarantee optimal culture conditions, culture-medium was replaced twice a week. Developmental stages (Lung stage (LuS), Early liver stage (eLiS), and Late liver stage (lLiS) schistosomula were examined by bright field microscopy using an inverted Axiovert 10 microscope (Zeiss) as previously published [10,27–29].

### Collection of *ex vivo* adult worms

3-4-week-old male NMRI mice were purchased from Envigo Germany and infected subcutaneously with approximately 200 *Schistosoma mansoni* cercariae (NMRI strain) per flank. After 6–7 weeks post-infection, mice were euthanized, and worms were collected from the portal and mesenteric veins via conventional perfusion [30]. Adult worms were placed in a petri dish with HM, washed once, and 10 worms/well were gently transferred into a 6-well plate with 2 ml HM supplemented with 20% HSe and rested for 24h at 37 °C, 5% CO_2_ before *in vitro* testing with sera and PAFAH.

### Recombinant protein production and purification and sequence alignment and homology modeling

The short-listed proteins identified as most likely active killing candidates (major urinary protein 10 (MUP10), fibroblast activation protein (FAP), and adiponectin) were cloned in pET28b without signal peptides using the NdeI and XhoI restriction sites and fusing the vector encoded N-terminal Histag. The recombinant proteins were expressed in *E. coli* BL21 strains and purified using metal affinity, ion exchange, hydrophobic interaction, and size exclusion chromatography. Recombinantly expressed in *E. coli* and purified mouse PAFAH (MsPAFAH) (PLA2G7, N-terminal His tagged, abx068540) and human PAFAH (HuPAFAH) (PLA2G7, N-terminal His tagged, abx068539) proteins were both purchased from Abbexa (Cambridge, UK), the purity checked, and dissolved in PBS, aliquoted at 1 mg/ml, and stored at -80 °C until use. The full-sequence recombinant mouse PAFAH that lacks enzymatic activity (InMsPAFAH) was generated with Abbexa Ltd.

Protein sequences and alignment were searched with UniProt and Clustal Omega tools, and homology modeling of both HuPAFAH and MsPAFAH structures and motif, domain, and active sites were performed using ExPASy (Swiss Bioinformatics Resources Portal) and AlphaFold2 modeling (UniProt) tools.

Structures of HuPAFAH (AA 54–427) and MsPAFAH (AA 22–426) (HUGO Gene name: PLA2G7) based on the 1.50 Å structure of HuPAFAH (3D59.pdb) and the AlphaFold2 predicted structure for MsPAFAH (available on UniProt entry Q13093) were visualized using UCSF ChimeraX 1.2.5. For the determination of the root mean square deviation (RMSD), the sequences of mouse (UniProt: Q60963) and human (UniProt Q13093) PAFAH were aligned using the Needleman-Wunsch algorithm in UCSF ChimeraX 1.10, and the RMSD values were calculated using the matchmaker function.

### *In vitro* assays with serum, PAFAH, and viability scoring

For *in vitro* culture of NTS, parasites were cultured in 96-well plates supplemented with 150 µl HM. Each well contained approximately 100 NTS. After 48h resting, wells with an average score ≥ 2.00 scoring points were selected for *in vitro* compound screening as previously established [27,28]. These were washed with HM to remove metabolites and waste generated during cultivation. Following the manufacturer’s instructions*,* PAFAH was dissolved in PBS and added at 1–50 µg/ml final concentrations. As a negative control, HM was adjusted to the same concentration of PBS as used for the PAFAH test. 20% mouse serum dissolved in HM (MSe) was used as a positive control to compare morphological effects between phenotypes and PAFAH. The worms were incubated at 37 °C and 5% CO_2_ for 168 h. PAFAH-induced morphological effects and viability scores were assessed after 24, 72, and 168 h using an inverted Axiovert 10 microscope (Zeiss, Germany) at 10x magnification. The viability of parasites in culture was scored as previously described [27,28]. Briefly, the scoring system is based on three main viability criteria: morphology, granularity, and motility, ranging from 0.00 to 3.00, with a score of 0.00 representing dead parasites and a score of 3.00 for entirely healthy, motile larvae. As the score represents an average of all NTS per well, subtle differences in viability were assessed by subdividing viability scores into 0.25 steps (e.g., 2.00, 2.25, 2.50, 2.75).

For long-term culture and advanced-stage development, 100 NTS were kept in 96-well plates and resuspended in HM supplemented with 20% human serum (HM + HSe). They were cultured at 37 °C, 5% CO_2_ until indicated time points post-transformation (p.t.). Developmental stages (lung stage schistosomula (LuS) and early (eLiS) and late liver schistosomula (lLiS)) were generated and characterized according to previously published morphological criteria [27,29,31].

For compound assessment, each well contained approximately 100 parasites with an average score ≥ 2.00 to be selected for screening [27,28]. After selection, juveniles were washed with HM to remove metabolites and waste generated during cultivation. PAFAH was dissolved in PBS and added in final concentrations of 1–50 µg/ml as indicated, and *in vitro* assays and viability scoring were performed as described above.

To inhibit PAFAH activity *in vitro*, we developed an inhibition assay. For this, Darapladib (DAR) (Bio-Techne, Cat# 6755), a selective and well-characterized inhibitor with high specificity for PAFAH [32,33] was prepared as 10 mM stock solution in dimethyl sulfoxide (DMSO) to be used at a final concentration of 5 nM diluted in HM. For the *in vitro* assay, DAR was pre-incubated with MSe or MsPAFAH (at respective concentrations as above) for 1 h at 37°C before incubation with NTS for 72 h.

### *In vitro* assays with adult worms

As previously described, *S. mansoni* adult worms of both sexes were flushed out from infected mice [28,34] and rested for 24h at 37 °C and 5% CO_2_. For the *in vitro* culture of adult couples, worms were cultured in 6-well plates supplemented with 2 ml HM for 10 worm couples or 10 females or males per well. PAFAH was dissolved in PBS and added in final 1–50 µg/ml concentrations as indicated. HM was used as a negative control with 20% MSe as a positive control. The worms were incubated at 37 °C and 5% CO_2_ for 72h; medium and compounds were exchanged every 24h. PAFAH-induced morphological effects were assessed every 24h using an inverted Axiovert 10 microscope (Zeiss) at 10x magnification.

Adult worm viability was scored as above and based on the recommendations by WHO-TDR [35,36], with the scores 3 (normal viability), 2 (reduced viability), 1 (minimal viability), and 0 (no movement within 30 sec was considered dead). Viability scores were subdivided into 0.25 steps. The scoring system is based on criteria like attachment to the well ground, granularity, and movement. The pairing status was observed to assess reproductive parameters for cultured worm couples, and eggs were counted daily. After 48h-72h, worms were either processed for confocal laser scanning microscopy (CLSM), scanning electron microscopy (SEM), and HPLC analysis or subjected to RNA extraction for quantitative real-time PCR (qPCR) analysis.

For phospholipid (PL) and free fatty acid (FFA) supplementation assays, *S. mansoni* adult worms were flushed out from infected mice and separated by sex, and 5 worms/well were plated as above in 2 ml DMEM (Gibco, ThermoFisher Scientific). Then, worms, treated with 15 µg/ml MsPAFAH and 20% MSe, were supplemented in parallel with 50 µM of C20:1/eicosenoic acid or 22:4/adrenic acid (Merck KGaA, Germany) to compensate the depletion of PC(16:0_20:1) and PE(18:0_22:4), or 18:0/stearic acid or 14:0 DMPC (1,2-dimyristoyl phosphatidylcholine) (Merck KGaA, Germany) as controls for 72h and viability was assessed as described above.

### Large-scale fractionation of mouse serum

Sera were harvested from naive mice derived from our in-house breeding facility or Envigo and diluted with PBS before fractionation using the Superdex 200 column (GE Healthcare Life Sciences). Collected fractions were pooled (F1, F2, F3, F4, and F5) according to dominant chromatographic peaks, and the volume was adjusted to the initial fractionated mouse serum volume and used at 20% for the NTS *in vitro* assay as described above.

### Mass spectrometry of active fractions and candidate selection strategy for identification of active components

For the identification of proteins present in the active fractions of mouse serum, the respective fractions were separated by SDS-PAGE. The resulting lanes of the SDS-PAGE were divided into 3–5 parts and sliced out of the gel. The proteins in the gel slices were reduced and alkylated before in-gel tryptic digest (using MS-grade trypsin, Promega, V511). The resulting peptides were extracted from the gel and applied to an UltiMate 3000 nano HPLC System, loading the peptide solution onto an Acclaim PepMap RSLC C18 trap column (Trap Column, NanoViper, 75 µm x 20mm, C18, 3 µm, 100 Å, Thermo Scientific) and separating the peptides on a PepMap RSLC C18 column (CoIP1: 75 µm x 50 mm C18, 2 µm, 100 Å, Thermo Scientific). Linear gradients from 4% (vol/vol) to 35% (vol/vol) acetonitrile with 0.1% formic acid were applied to elute the peptides, which were subsequently analyzed in an Orbitrap Q Exactive plus (Thermo Scientific) mass spectrometer. Full scans of collision-induced dissociation MS^2^ (scan 2 mode) of the most intense ions were recorded throughout the elution gradient.

The mass spectrometry data from each sample were searched against the Swiss-Prot mouse database downloaded from UniProt using the Sequest HT Algorithm implemented into the “Proteome Discoverer 1.4” software (Thermo Scientific). The search was limited to peptides containing a maximum of two missed cleavage sites and a peptide tolerance of 10 ppm (parts per million) for precursors and 0.04 Da for fragment masses. Proteins were identified with at least one unique peptide with a target discovery score of higher than one. For further evaluation, three independent datasets resulting from biological replicates were compared.

The overall strategy was to identify molecules responsible for the anti-schistosomal activity present in fraction F2, displaying the strongest parasite-killing activity during serum fractionation. As fraction F2 still retained substantial anti-schistosomal activity even after prolonged heat treatment (60 min), we reasoned that the active factor(s) responsible for the phenotype must be present in both untreated F2 and heat-treated F2 (60 min). Therefore, we focused on overlapping proteins shared between these two active conditions. This filtering step yielded 69 candidate proteins, which were considered the most likely to represent heat-stable and active components contributing to the observed phenotype.

Following identification of proteins in the active fraction, we applied additional filtering criteria based on Gene Ontology annotations to prioritize candidates most consistent with the expected biological properties of the active factor. These criteria included: 1) Type and state: Extracellular localization or secretion, consistent with a serum-derived protein containing factor; 2) Function and activity: Protein-, lipid-, phospholipid-binding activity and calcium-dependent, commonly associated with binding to extracellular and structural components of the schistosome worms and the osmotic sensitivity of the bioassays; and 3) Induced signaling and pathways: Linked to protein- and lipid-metabolism due to key role of schistosome tegument- and membrane-enriched lipid, phospholipid disruption signaling for the survival of the parasite. These criteria helped prioritize candidate molecules likely to interact with parasite membranes or lipid structures and signaling relevant for schistosome viability.

### Confocal laser scanning microscopy (CLSM)

For morphological analysis by CLSM, worms from different runs were fixed and stained for 30 min with carmine red (CertistainH; Merck, Germany) as described before [37,38]. After treatment, the worms were fixed in AFA (95% alcohol, 3% formalin, and 2% glacial acetic acid) and stained with 2.5% hydrochloric carmine (Certistain, Merck). The specimens were preserved as whole mounts in Canada balsam (Merck) on glass slides. Stained worms were examined on an inverse CLSM (Confocal Olympus FV3000). Carmine red was excited with an argon-ion laser at 488 nm. Laser power and gain and offset of all photomultiplier tubes (PMTs) were optimized to minimize possible bleaching effects and complete range intensity coding using the CLUT function (color look-up table) of the Olympus FV3000 software. Background signals and optical section thickness were defined by setting the pinhole size to airy unit 1.

### Scanning electron microscopy (SEM)

After culture, parasites were collected in protein low-binding Eppendorf tubes, fixed in 4% glutaraldehyde/ 0.2 M cacodylate, and washed with 0.4 M saccharose/ 0.2 M cacodylate. Following fixation, worms were treated with 1% osmic acid/ 0.1 M cacodylate and washed for a second time in distilled water, followed by ethanol dehydration at 30%, 50%, and 70% concentration. The next day, dehydration in 80%, 90%, 96%, 99.8%, and 100% ethanol continued. After all, samples were air-dried in a CPD030 critical point dryer (BAL-TEC AG; Balzers, Liechtenstein), mounted, and coated with gold sputter before imaging with a Gemini DSM 982 (Carl Zeiss Microscopy; Oberkochen, Germany), operating at 3 kV.

### MALDI-MS imaging

The sectioning protocol for the samples was adapted from Mokosch et al. [39]. Instead of 15 µL, 30 µL of gelatine solution were used. The sections of the male samples had a thickness of 20 µm, and the female sections were 16 µm thick. The samples were measured with an atmospheric-pressure MALDI imaging ion source (AP-SMALDI5 AF, TransMIT GmbH) coupled to an orbital trapping mass spectrometer (Q Exactive HF, Thermo Fisher Scientific). Measurements were performed in positive ion mode using a mass range of m/z 250–1000, a mass resolution of R = 240,000 @ m/z 200, and a spatial resolution (i.e., step size) of 7 µm. Internal mass calibration was achieved using the lock-mass feature of the orbital trapping mass spectrometer, resulting in a mass accuracy of < 3 ppm. For data analysis, raw data of all measurements were stitched together and converted to imzML imaging data file format. Annotation of lipid signals ([M + H]^+^, [M + Na]^+^, [M + K]^+^) was performed by Metaspace, a fully automated metabolite annotation platform for imaging data [40], using the databases of HMDB-v4, LipidMaps-2017-12-12 and SwissLipids-2018-02-02 and a ± 3 ppm m/z window.

For semi-quantification, regions of interest (ROI) comprising the tissue of each worm were defined. Total ion count (TIC) normalized images of annotated lipids (+/- 3 ppm) containing the ROIs of all samples were exported from MIRION software in CSV-Format [41]. In Matlab, the intensity sum of each lipid signal was calculated per ROI and divided by the ROI pixel number for each sample.

### Extraction and quantitative analysis of phospholipids

Phospholipids were extracted from cell pellets by the sequential addition of PBS pH 7.4, methanol (spiked with internal standards), chloroform, and saline (final ratio 14:34:35:17). The organic layer was evaporated using an Eppendorf Concentrator Plus system (Hamburg, Germany; non-polar phase: high vapor pressure application mode), stored at -20°C, and dissolved in methanol before analysis [42,43]. Internal standards: 1-pentadecanoyl-2-oleoyl(d7)-sn-glycero-3-phosphocholine [PC(15:0_18:1-d7), Avanti Polar Lipids, Alabaster, AL] and 1-pentadecanoyl-2-oleoyl(d7)-sn-glycero-3-phosphoethanolamine [PE(15:0_18:1-d7), Avanti Polar Lipids].

Phospholipids were separated on an Acquity UPLC BEH C8 column (130 Å, 1.7 µm, 2.1 × 100 mm, Waters, Milford, MA) using an ExionLC AD UHPLC system (Sciex, Framingham, MA) and detected by a QTRAP 6500^+^ mass spectrometer (Sciex) equipped with an IonDrive Turbo V Ion Source and a TurboIonSpray probe for electrospray ionization [43]. Chromatographic separation was performed at 45°C and a flow rate of 0.75 ml/min using mobile phase A (water/acetonitrile 90/10, 2 mM ammonium acetate) and mobile phase B (water/acetonitrile 5/95, 2 mM ammonium acetate). The gradient was ramped from 75 to 85% B over 5 min and then increased to 100% B within 2 min, followed by isocratic elution for another 2 min. Optimized MS source and compound parameters for PC and PE are shown in Table 1. The QTRAP 6500^+^ system was operated in negative ionization mode using scheduled multiple reaction monitoring (MRM). The transitions from [M-H]^-^ to both fatty acid anions were monitored [42,43] to detect PE and PC species. The instruments were operated by Analyst 1.7.1 (Sciex), and the mass spectra were processed by Analyst 1.6.3 (Sciex).

**Table 1:** Source and compound parameters.

Parameters	PC (diacyl)	PE (diacyl- and ether-)
Curtain gas (CUR)	40 psi	40 psi
Collision gas (CAD)	medium	medium
Ion spray voltage (IS)	-4,500 V	-4,500 V
Heated capillary temperature (TEM)	350°C	650°C
Sheath gas pressure (GS1)	55 psi	55 psi
Auxiliary gas (GS2)	75 psi	75 psi
Declustering potential (DP)	-44 V	-50 V
Entrance potential (EP)	-10 V	-10 V
Collision energy (CE)	-46 eV	-38 eV
Collision cell exit potential (CXP)	-11 V	-12 V

Combined peak areas from the transitions to the two fatty acid anions were calculated for the quantitative analysis of PE and PC. To calculate absolute lipid quantities, signals were normalized to a subgroup-specific internal standard [PE(15:0_18:1-d7) or PC(15:0_18:1-d7)]. Relative intensities of phospholipids are given as a percentage of all species detected in the corresponding phospholipid subclass (= 100%). Cellular proportions of phospholipid subgroups were calculated by summarizing the relative intensities of individual species.

### ELISA and PAFAH protein activity assay

According to the manufacturer’s instructions, PAFAH concentrations in infected and non-infected mouse serum were determined using commercially available enzyme immunoassay kits (Abbexa). PAFAH enzyme activity in different sera and of the recombinantly expressed protein MsPAFAH (44 µg/ml) was assessed using the PAF Acetylhydrolase Assay Kit (Cayman Chemical) according to the instructions provided by the manufacturer and with HuPAFAH as positive control.

### RNA isolation and RT-qPCR analysis

Freshly perfused or *in vitro*-cultured worm couples were separated by gender, and 5 worms per gender were incubated in different culture conditions in du-/triplicate. After the indicated time points, RNA was extracted from male and female worms using the Monarch Total RNA Miniprep Kit (New England Biolabs) according to the manufacturer’s protocol. Reverse RNA transcription in cDNA was performed using the Quantitect RT-Kit (QIAGEN). Expression levels of the *S*. *mansoni* orthologs of the stem cell markers *nanos-1* (Smp_055740) and *fgfr1* (Smp_157300) and apoptosis *bcl2* (Smp_072180) were determined by RT-qPCR using the KAPA SYBR FAST qPCR Master Mix (2X) Kit (Sigma-Aldrich). All samples were pipetted in technical triplicates. Ct values were normalized against the geometric mean of the reference gene *letm1* (Leucine zipper/EF‑hand‑containing transmembrane protein 1) (Smp_065110), selected based on stable expression in both sexes [44]. Relative expression levels were calculated by the delta-delta Ct method [45] or by expressing the data as n-fold difference by the formula: relative expression = 2^−delta Ct^ × f, with f = 1 as an arbitrary factor. The following primers were used, which were confirmed by test qPCRs to have efficacies between 0.9–1:

LETM1_ forward (fw) 5’-CGTGGAATGCGTTCAGTTGG-3’,

LETM1_reverse (rev) 5’-GAAGCTGATGGAGGTAATTGAG-3’;

Nanos-1_fw 5’-ACTTGTCCATTATGCGGTGCT-3’,

Nanos-1_rev 5’-GGTTCCAACAAACCAGCTTCA-3’;

Bcl2_fw 5´-TCTTCATGATGGTTGGTCTGGA-3’,

Bcl2_rev 5´CCGACAAGAGCAGCTAAACC-3’;

FGFR1_fw 5´-CACAGAAGGAGATGTGTCTGAA-3’

FGFR1_rev 5´-TTCCCGTAAGGAGCATATTCCA-3’.

### Statistical analysis

The data were analyzed using the software Prism 10, Version 10.1.2 (GraphPad Software, LLC, San Diego, CA, USA). Quantitative data are expressed as mean ± SEM and comparative analysis among was conducted using one-way and two-way ANOVA followed par multiple comparison tests (> 2 groups) or Mann-Whitney U test (= 2 groups). Statistical significance was accepted when *p* < 0.05.

## Results

### Fractionation of mouse serum reveals PAFAH as an active anti-schistosomal multi-stage enzyme

**Fig 1:**
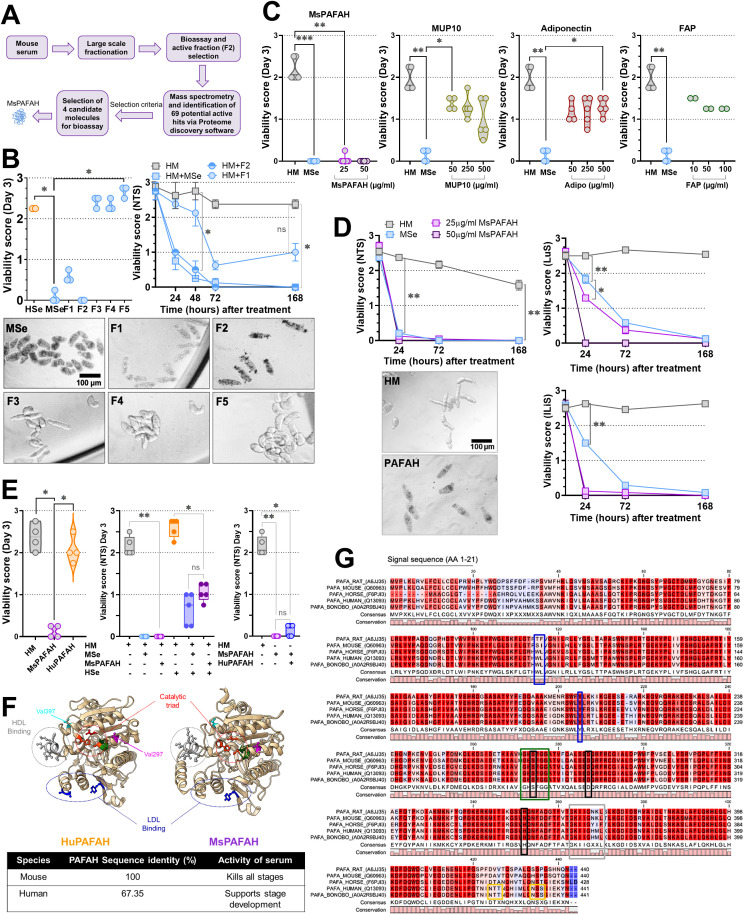
Large-scale fractionation of mouse serum reveals MsPAFAH as an active anti-schistosomal multi-stage enzyme. **(A)** Schematic procedure of large-scale fractionation of mouse serum and identification of candidate molecules and mouse platelet-activating factor-acetylhydrolase (MsPAFAH). **(B)** Left panel: Effect of human (HSe) and mouse (MSe) sera and fractions at 20% on the viability of newly transformed schistosomulae (NTS) at day 3 (left panel). A representative picture is shown on the lower panel. Right panel: Time-dependent schistosome killing activity of mouse serum active fractions F1 and F2 in culture. **(C)** Concentration-dependent effect of mouse serum selected recombinant expressed candidate molecules, MsPAFAH, major urinary protein 10 (MUP10), fibroblast activation protein (FAP) and Adiponectin as compared to mouse serum (MSe) and HM control on NTS at day 3. **(D)** Time-dependent effect of MsPAFAH on different *S. mansoni* developmental larval stages (skin (NTS), lung (LuS), late liver juvenile (lLiS)). **(E)** Left panel: Comparison of the effect of recombinant mouse (MsPAFAH) and human PAFAH (HuPAFAH) on NTS at day 3. Middle panel: Schistosome killing activity of mouse serum (MSe) and PAFAH (MsPAFAH) in the presence human serum (HSe). Right panel: Comparative analysis of schistosome killing activity of MsPAFAH alone or in presence of human PAFAH (HuPAFAH). **(F)** Structural comparison and homology modeling of HuPAFAH and MsPAFAH. Both structures have a classic α/β serine hydrolase fold and contain a catalytic triad (Ser273, His351, and Asp296 in HuPAFAH; Ser272, His350, and Asp295 in MsPAFAH (in red) and HDL/LDL (Low/high-density lipoproteins) binding sites (blue) (upper panel) with sequence identity and killing properties (lower panel). **(G)** Alignment of selected PAFAH amino acid sequences (HUGO gene name: PLA2G7). Sequences were retrieved from UniProt (rat: A6JJ35; mouse: Q60963; human: Q13093; bonobo: A0A2R9BJ40; horse: F6PJI3) and aligned using Clustal Omega default settings. Green box: GXSXG motif; Black box: catalytic triad corresponding to the structure shown in Fig 1F; Orange box: predicted NXS/T N-glycosylation sites; Grey box: HDL-binding region shown in Fig 1F; Blue boxes: residues involved in LDL binding shown in Fig 1F. Data information and statistics: Results are representative of at least three independent experiments and are expressed as means ± SEM. Asterisks show significant statistical differences analyzed using Kruskal–Wallis one-way ANOVA followed by a Dunn’s multiple comparison test (B, C, E) and two-way ANOVA followed by Tukey’s multiple comparison test **(D)**. *P < 0.05; **P < 0.01; ***P < 0.001.

Humans are known to be the primary definitive host for *S. mansoni*, while the mouse is the most widely used experimental laboratory host. Recently, we observed that when human serum (HSe) was replaced by mouse serum (MSe) in our cell-free *ex vivo*-culture system for molecule and drug testing [27,28], newly transformed schistosomulae (NTS) as well as developing advanced larval stages died within 72 hours [10], suggesting that MSe has soluble serum factor(s), which are detrimental for survival and development of mammalian host dwelling stages of *S. mansoni* [27,28]. To identify the schistosomicidal serum factor(s), we performed a large-scale fractionation of MSe using a Superdex 200 size exclusion column, pooled 35 fractions into 5 subfractions (F1-5), and tested these in the NTS bioassay (Figs 1A and S1A). Our *ex vivo* study revealed two fractions (F1 and F2) with NTS killing effect, whereby only fraction F2 very efficiently killed NTS comparable to the schistosomicidal effect of 20% MSe (Fig 1B, left panel). Additional analyses showed that F1, in contrast F2 displayed less consistent and scalable activity (Fig 1B, right panel).

Furthermore, heating at 99°C for 60 min and Pronase E treatment of MSe and fraction F2, did not reliably abolish NTS killing capacity of F2 (S1B Fig). Specifically, treatment of fraction F2 with proteinase K, a serine protease, resulted in a substantial, although not full, reduction of the schistosome-killing activity (S1B Fig), indicating that the NTS killing effects could be due to a protein, including lipoproteins and phospholipoproteins. Next, to identify the protein(s) responsible for the killing activity, we analyzed the different killing and non-killing fractions using comparative mass spectrometry. Afterward, the subtraction of proteins found in the inactive fractions from the set of proteins identified in the active heated and native F2 fractions allowed the restriction of the effective proteins in an initial step to 69 potential candidates for the active killing component in MSe (S1C Fig).

From these 69 potential candidates, four (PAFAH, major urinary protein 10 (MUP10), fibroblast activation protein (FAP), and adiponectin) were chosen for recombinant expression based on different criteria as detailed in the methods. All four purified candidate proteins were tested for their effect on the viability of NTS as compared to MSe and HSe (Fig 1C). Interestingly, mouse PAFAH (MsPAFAH) had the strongest viability-reducing effect on NTS after 72 h with a significant impact visible at a concentration of 15 µg/mL with a lethal effect at 25 µg/mL (Figs 1C and S1D). The enzymatic activity of the recombinant MsPAFAH was confirmed using a commercial assay and, was less active when compared to PAFAH activity of MSe correlating with the slightly lower killing efficiency *in vitro* (S1E Fig) as compared to MSe. We also observed killing properties for the other proteins, however, at comparably higher concentrations of 500 µg/ml (Fig 1C) with no effect at lower concentrations (less than 10 µg/ml) (S1F Fig). Thus, we moved on with investigating in more detail MsPAFAH, which showed the closest schistosomicidal profile with mouse serum. We next investigated the killing impact of MsPAFAH on more advanced *in vitro*-generated *S. mansoni* larval stages (lung stage (LuS), late liver juvenile (lLiS)), critical for host reinfection. As for MSe, almost all mammalian host-dwelling stages of *S. mansoni* were killed within 72 h (Fig 1D). Of note, LuS was less affected by MSe and MsPAFAH at 72 h as compared with NTS and lLiS, the closest developmental stage to the adult worm in human schistosomiasis (Fig 1D). Interestingly, the killing efficiency of MsPAFAH of NTS was strongly impaired in presence of the selective PAFAH inhibitor Darapladib (DAR) [32,33], confirming that parasite killing depends on PAFAH enzymatic activity (S1G Fig). Inhibition by DAR also reduced the killing activity observed with whole mouse serum (S1G Fig), indicative of potential further killing component(s) in MSe. To further confirm the enzymatic activity of MsPAFAH in the schistosome killing, we generated a catalytically inactive recombinant InMsPAFAH variant. In contrast to the enzymatically active MsPAFAH protein, the inactive mutant failed to induce parasite killing (S1H Fig) demonstrating that the observed schistosomicidal activity depends on PAFAH enzymatic function rather than nonspecific protein toxicity.

Since humans are the major definitive host of *S. mansoni*, we next investigated whether MSe and MsPAFAH remain active under host-permissive serum conditions. We then performed a co-application experiments in which parasites were cultured in the presence of human serum (HSe) or human PAFAH (HuPAFAH), which normally supports parasite survival and development, and then treated with either MSe or MsPAFAH. Interestingly, we observed no killing effect by HuPAFAH as compared to MsPAFAH (25 µg/mL each) (Fig 1E, left panel), reflecting our previous findings that HSe did not only fail to kill NTS but also promoted their development *in vitro* to juvenile worms [10]. In addition, both MSe and recombinant MsPAFAH retained strong schistosomicidal activity even in the presence of HSe, although with slightly less efficiency at day 3 as compared with treatments performed in the absence of HSe (Fig 1E, middle and right panels). These results demonstrate that the activity of MsPAFAH is not suppressed in a human-serum environment. Importantly, sequence alignment and homology-modeling of HuPAFAH and MsPAFAH structures [46, 47] [48] revealed that while the Ser/Asp/His catalytic triad of serine hydrolases as well as other residues (Val297 and Val397) important for activity are perfectly conserved, there are major differences in the LDL binding region (Fig 1F and 1G), relevant for the enzymatic activity. In addition, their amino acid sequences showed only 67.35% overall identity, including the 21 amino acid signal sequences (Fig 1F and 1G).

Collectively, our data so far indicate that MsPAFAH represents one of the strongest schistosomicidal components present in MSe and is responsible for the efficient killing of different stages of *S. mansoni ex vivo*.

### PAFAH is upregulated during *S. mansoni* infection in mouse serum and reduces the reproductivity of adult worms

**Fig 2:**
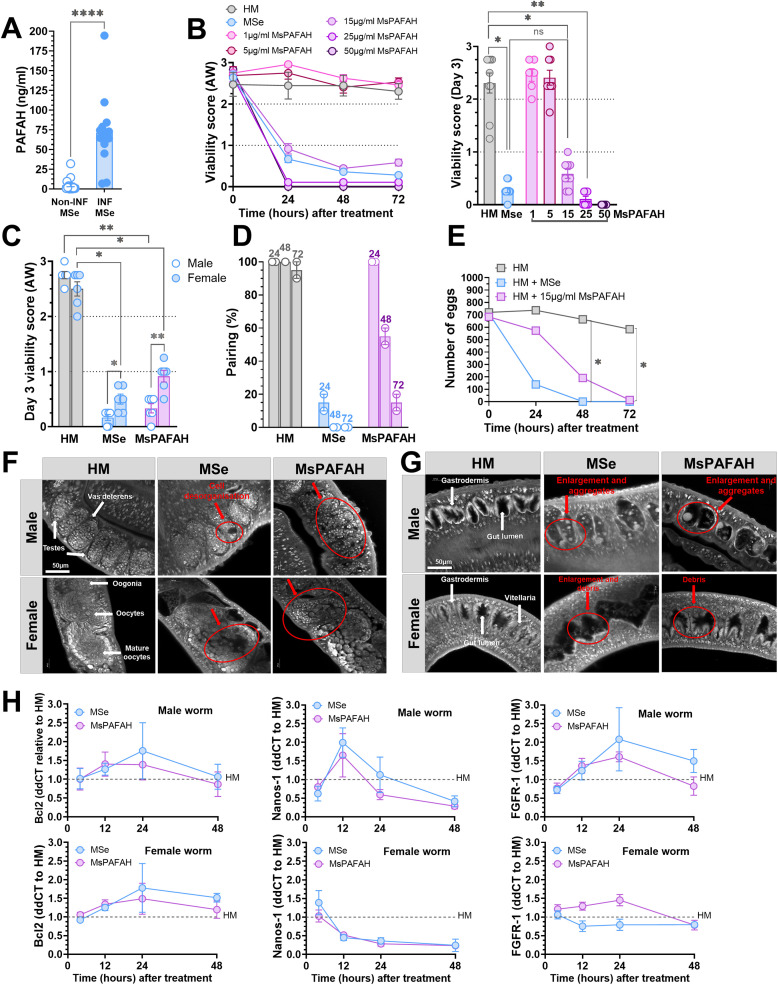
MsPAFAH is upregulated during *S. mansoni* infection in serum and reduces the reproductivity of adult worms. **(A)** Serum levels of MsPAFAH in schistosome-infected and non-infected mice. **(B)** Concentration- and time-dependent effects of MsPAFAH on ex-vivo adult worms recovered from infected mice. **(C)** Effects of MsPAFAH and MSe on male compared to female adult worms at day 3. **(D)** Impact of MsPAFAH and MSe on male and female adult worm pairing. **(E)** Number of eggs released by female adult worms over time following treatment with MsPAFAH and MSe. **(F)** CLSM-analysis of the effect of MsPAFAH and MSe treatment on gonadal tissues of male and female adult worms at day 3. White arrows show structure localization. Red arrows show affected areas and tissue disorganization by MsPAFAH and MSe. **(G)** CLSM-analysis of the effect of MsPAFAH and MSe treatment on intestinal tissues of male and female adult worms at day 3. White arrows show structure localization. Red arrows show affected areas and aggregate and debris formation by MsPAFAH and MSe. (H) qPCR analysis of MsPAFAH- and MSe-driven regulation of the expression of anti-apoptotic and gonadal stem cell proliferation- and differentiation-related genes in male and female adult worms. Data information and statistics: Results are representative of at least three independent experiments (5-10 worm pairs per condition) and are expressed as means ± SEM. Asterisks show significant statistical differences analyzed using Kruskal–Wallis one-way ANOVA followed by Dunn’s multiple comparison test (A, B, C) and two-way ANOVA followed by Tukey’s multiple comparison test **(E)**. *P < 0.05; **P < 0.01; ****P < 0.0001.

To investigate whether PAFAH is regulated *in vivo* during schistosome infection as an indication of its potential role in host defense, we infected mice for 7 weeks with *S. mansoni* and evaluated serum levels of PAFAH in infected and naïve control animals. As depicted in Fig 2A, the infection markedly increased MsPAFAH levels in the serum, although both infected and uninfected serum displayed higher PAFAH activity. Having shown that MsPAFAH profoundly influences the viability of *ex vivo*-generated *S. mansoni* developmental stages and is upregulated during infection, we examined the impacts of MSe and MsPAFAH on adult worms isolated from infected mice. For this, mature *S. mansoni* worms of both sexes were flushed from infected mice and cultured *ex vivo* in couples or individually (Fig 2B-2C). Interestingly, MSe and MsPAFAH similarly reduced the viability of worm couples in a time- and concentration-dependent manner, with a significant effect of MsPAFAH at 15 µg/mL within 48–72 h (Fig 2B). Importantly, although both sexes of *S. mansoni* were heavily affected after 72 h of treatment with MSe and MsPAFAH, we observed a sex-biased viability effect with males being more susceptible to the treatment (Fig 2C). Similar sex-differences were previously reported for schistomicidal small molecules, such as PZQ [49,50].

Human schistosomiasis mainly results from the immunopathology induced by host responses to deposited eggs in tissues, such as in the liver [51,52]. Unlike other trematodes, successful egg production depends on worm pairing stability [51,52]. We thus conducted a pairing stability assay in the presence of MSe and MsPAFAH (Fig 2D). We observed an 80% reduction of pairing stability at 24 h post-treatment (p. t.) with MSe. As expected, MsPAFAH also affected the pairing stability of *S. mansoni*; however, the effect was a bit delayed (Fig 2D). Accordingly, we noted a significant reduction in the number of eggs deposited in culture at 48 h post-treatment for both MSe and MsPAFAH, with a complete arrest in egg production at 72 h compared to the non-treated control in hybridoma medium (HM) (Fig 2E). To investigate whether these observations were related to morphological defects in the parasite’s reproductive system, MSe- and MsPAFAH-treated worms were fixed for subsequent confocal laser scanning microscopy (CLSM) analysis. We detected morphological abnormalities at the organ level (Fig 2F-2G), as representative examples of affected structural phenotypes complementing the quantitative functional assays and measurements observed across multiple worms. In untreated control males, testes were composed of several testicular lobes containing numerous spermatogonia and spermatocytes in different stages of maturation (Fig 2F, upper panel left). Physiologically, spermatogenesis is located at the dorsal part of the lobes with large round spermatogonia and expires at the ventral part, indicated by smaller elongated mature sperms (spermatozoa) [37,38]. After MSe and MsPAFAH treatment, these lobes appeared disorganized and showed porous areas, creating a Swiss*-*cheese-like tissue pattern (Fig 2F, upper panel, middle and right). Nevertheless, the seminal vesicle still contained some spermatocytes of unknown quality. In control females, the small immature oogonia were detected within the anterior part of the ovary, and bigger mature oocytes were found within the larger posterior part [37,38]. We observed that MSe and MsPAFAH also disrupted the structure of the ovaries (Fig 2F, lower panel, middle and right). They appeared disorganized and shrunken, and oogonia of smaller size and mature oocytes were poorly separated from each other. Unlike control ovaries, hole-like areas were found within the ovary, indicating a lack of cell-based staining. In contrast, in the absence of MSe and MsPAFAH treatment, the vitellarium of control females was found to be tightly packed with cells, which interlocked with the cell rows of the opposite side in a zipper-like arrangement as previously reported [37,38].

We further evaluated the internal structure damage of the digestive organs of treated worms (Fig 2G). Whereas the gastrodermis of control males appeared as a thick, continuous layer of syncytial cells [53,54], MSe and MsPAFAH-treated worms showed an enlarged gut lumen and degradation of the gastrodermis, which led to the accumulation of degraded tissue and particle aggregates of remarkable size (Fig 2G, upper panel, middle and right). Especially, the gastrodermis appeared to be detached from the adjacent tissue layer and completely collapsed in some compartments. Although females appeared less affected by the internal destruction of the digestive organs, reflecting the viability assessment, enlargement and debris were noted following MSe and MsPAFAH treatment (Fig 2G, lower panel, middle and right).

In summary, treatment with MSe and MsPAFAH led to a dramatic reduction of schistosomal eggs. This might be related to the structural disruption following apoptotic signaling and disintegration of stem and reproductive cells in the ovary and testes, as reported elsewhere [55,56]. Therefore, we investigated the regulation of genes related to apoptosis (*bcl2*) and gonadal stem cell proliferation, stability, and differentiation (*nanos1, fgfr1*) important in female reproduction [55,56]. In line with the degraded gonadal cells noted by CLSM, we observed a downregulation of the expression of *nanos1* and, to a lesser extent, *fgfr1* in both male and female adult worms in the presence of MSe and MsPAFAH (Fig 2H). In contrast, there was an increase in the expression of anti-apoptotic *bcl2* after 12–24 h following MSe and MsPAFAH treatment, which remained after 24 h and 48 h only in female worms, possibly as a sign of better survival and active apoptosis regulating mechanisms as compared to males (Fig 2H). Of note, except for *fgfr1* regulation in female worms, both MSe and MsPAHAH had comparable effects overall.

Taken together, these data suggest that, in mice, PAFAH might be a potent host defense enzyme regulated during schistosome infection with the capacity to severely reduce the parasite viability and reproductive activity.

### MsPAFAH manipulates the schistosome phosphatidylethanolamine (PE) and phosphatidylcholine (PC) composition and distribution

**Fig 3:**
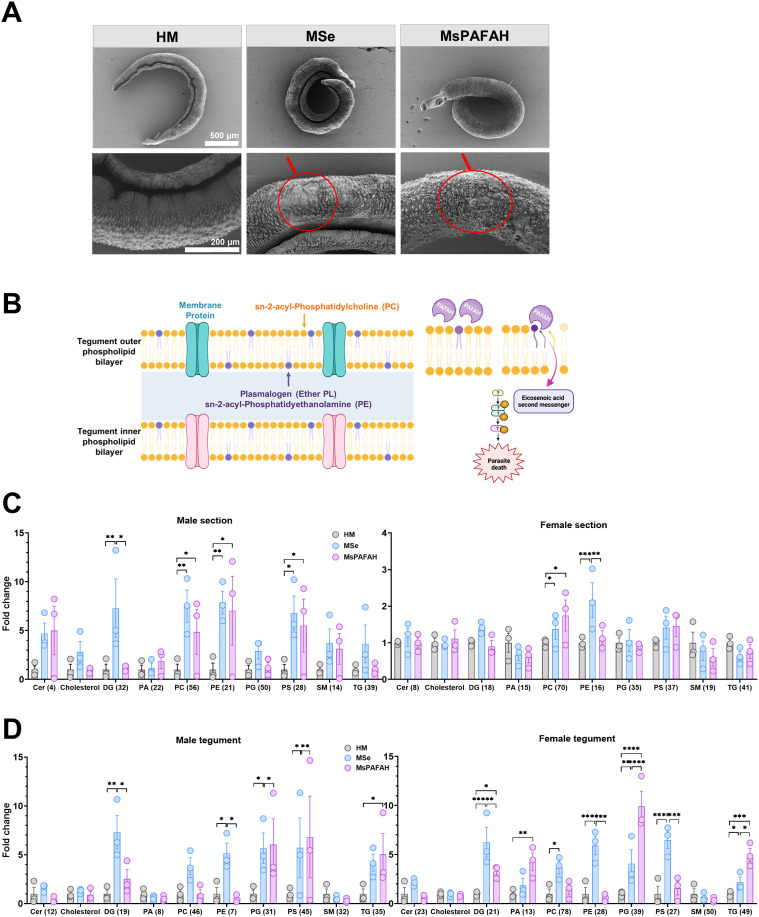
MsPAFAH remodels the schistosome phospholipids PE and PC composition and distribution. **(A)** SEM analysis of the effect of MsPAFAH and MSe treatment on ex-vivo adult worm tegument at day 3. Red arrows show disorganized tegument tissue layers following MsPAFAH and MSe treatment. **(B)** Schematic representation of schistosome tegumental phospholipid layers (left panel) and investigation hypothesis of the effect of MsPAFAH and potential downstream signaling. Created in BioRender. https://BioRender.com/3oyh1uf. **C)** MALDI-MSI analysis of fold change in the composition of male (left panel) and female (right panel) adult worm phospholipids and lipids following MsPAFAH and MSe treatment as compared to HM control. Numbers in brackets are the number of lipids detected and analyzed. Cer: Ceramides; DG: Diacylglycerols; PA: Phosphatidic acids; PE: Phosphatidylethanolamines PC: Phosphatidylcholines; PG: Phosphatidylglycerols; PS: Phosphatidylserines; SM: Sphingomyelins; TG: Triglycerides. **(D)** MALDI-MSI analysis of MsPAFAH- and MSe-induced fold change of the major male (left panel) and female (right panel) schistosome tegument phospholipid classes. Numbers in brackets are the number of lipids analyzed. Data information and statistics: Results are representative of at least two to three independent experiments (5 worms) and are expressed as means ± SEM. Asterisks show significant statistical differences analyzed using Kruskal–Wallis two-way ANOVA followed by Tukey’s multiple comparison test. *P < 0.05; **P < 0.01; ***P < 0.001.

Having characterized the internal structural degradation induced by PAFAH present in MSe, we next investigated whether the observed phenotypes could originate from an effect on schistosomal surface membranes and potential apoptotic signaling as reflected by massive granulation in parasite tissues in the presence of MsPAFAH and MSe. Thus, we conducted scanning electron microscopy (SEM) imaging of formalin-fixed adult worms following treatment with MSe and MsPAFAH. Compared to the untreated HM control, we observed, in the treated groups, contracted worms at lower magnification (Fig 3A, upper panel, middle and right) and, at higher magnification, damaged and disorganized tegumental structures at 72 h p. t. (Fig 3A, lower panel, red circles).

The tegument of schistosomes is essential for the survival of the parasite [50]. As presented in Fig 3B, the tegument of *S. mansoni* is composed of a syncytial structure consisting of two (outer and inner) closely apposed phospholipid (PL) bilayers that form the interactive surface with the host. PAFAH hydrolyses PL with short-chain acyl groups (n < 8) at sn-2 position (sn-2-acyl-PLs e.g. platelet-activating factor (PAF)) as well as oxidized PLs with longer acyl chains having aldehyde, carboxylic acid, hyperperoxid or epoxy groups, thereby generating lyso-PAF, lyso-PLs [57], free fatty acids and lipid mediators [58]. In addition, PAFAH has a transacetylase activity contribtuting to PAF diversity [58]. Therefore, we hypothesized that MsPAFAH binds to, hydrolyzes, and disrupts worm tegument sn-2-acyl-PLs, leading to worm death, possibly involving oxidized PLs in schistosomes, whose presence is controversial [59]. We thus first analyzed the lipid profile of male and female worm sections (Fig 3C) and teguments (Fig 3D) using untargeted metabolipidomics and imaging. The matrix-assisted laser desorption/ionization mass spectrometry imaging (MALDI-MSI) surprisingly revealed an upregulation of PL levels in response to both MSe and MsPAFAH after 72 h of treatment, both in the body (Fig 3C) and in the tegument (Fig 3D) of the worms, with a significant change of PE and PC, the major PLs in schistosomes [50] and less abundant lipids such as the triglycerides (TG).

These data demonstrate a remodeling of the distribution and composition of PLs, mainly of the PE and PC species, upon MsPAFAH and MSe treatment.

### MsPAFAH, but not HuPAFAH, depletes specific unsaturated PC and PE species of schistosomes while increasing overall diacyl- and lyso-phospholipid levels

To gain deeper insights into how MSe and MsPAFAH remodel adult worm PC and PE species, we performed quantitative metabololipidomics using a more sensitive ultra-performance liquid chromatography-tandem mass spectrometry (UPLC-MS/MS) system [59,60]. Profiling of PC and PE species revealed that male worms have higher total diacyl-PC and diacyl-PE levels than female worms (S2A Fig), consistent with their larger body size. In addition, the fatty acid compositions in diacyl-PC and diacyl-PE significantly differed between sexes (S2B Fig). For example, the predominant PC species, PC(palmitate (16:0)_eicosenoate (20:1)), was considerably higher in males than females (42.3% vs. 26.4%), whereas PE(stearoyl (18:0)_adrenate (22:4)) was more abundant in females (42.4% vs 36.3%) (S2C Fig), confirming previously reported baseline sex differences in *S. mansoni* membrane PL profiles [50].

**Fig 4:**
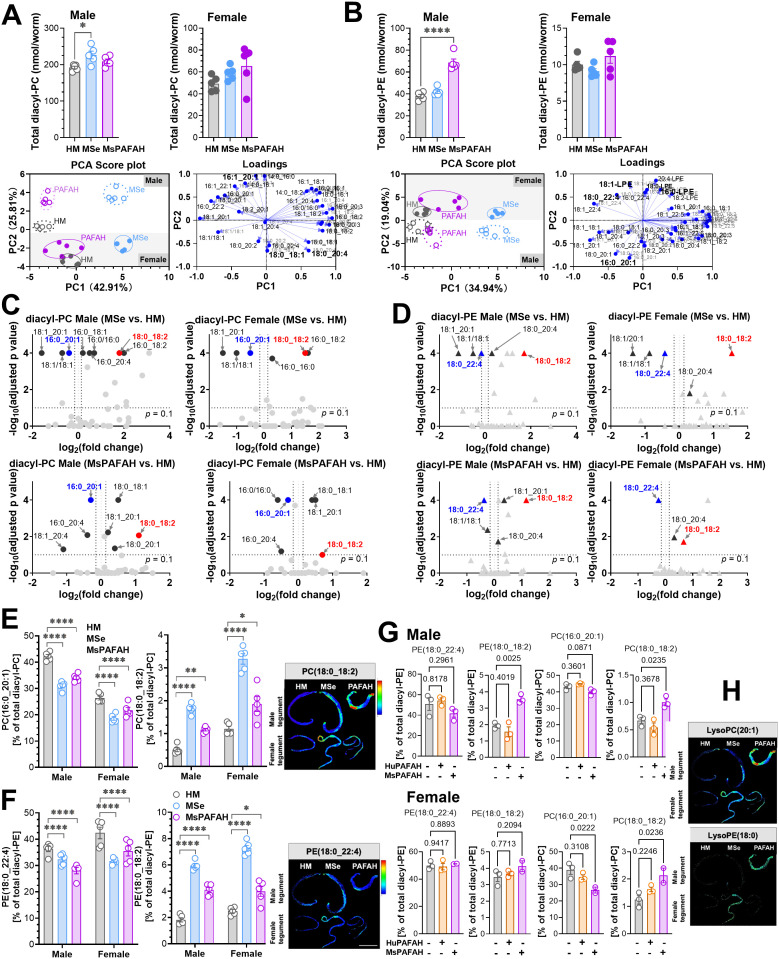
MsPAFAH, but not HuPAFAH, depletes specific unsaturated PC and PE species of schistosomes while increasing overall diacyl- and lyso-phospholipid levels. **(A)** UPLC-MS/MS analysis of extracted diacyl-phosphatidylcholine (PC) from male and female adult worms (upper panel); and PCA score plots clustering sex-dependent effects (lower left panel) and corresponding loading plots (lower right panel) following MsPAFAH and MSe treatment. Graphs show the total diacyl-PC amount (pmol/worm, upper panel); PCA plots were generated using the relative intensities of individual diacyl-PC species (% of total diacyl-PC). **(B)** UPLC-MS/MS analysis of extracted diacyl-phosphatidylethanolamine (PE) from male and female adult worms (upper panel) and PCA score plots clustering sex-dependent effects (lower left panel) and corresponding loading plots (lower right panel) following MsPAFAH and MSe treatment. Graphs show the total PE amount (pmol/worm, upper panel); PCA plots were generated using the relative intensities of individual diacyl-PE species (% of total diacyl-PE). **(C)** Volcano plots showing the differentially regulated diacyl-PC species (relative intensities, % of total diacyl-PC) upon MsPAFAH and MSe treatment in male and female worms. **(D)** Volcano plots showing the differentially regulated diacyl-PE species (relative intensities, % of total diacyl-PE) upon MsPAFAH and MSe treatment in male and female worms. **(E)** Proportion of the downregulated PC(16:0_20:1) and upregulated PC(18:0_18:2) species following MsPAFAH and MSe treatment. **(F)** Proportion of the downregulated PE(18:0_22:4) and upregulated PE(18:0_18:2) species following MsPAFAH and MSe treatment. The right panels show MALDI-MS imaging of the PC (18:0_18:2) and PE(18:0_22:4) species. **(G)** Proportion of the downregulated PC(16:0_20:1) and PE(18:0_22:4), and upregulated PC and PE(18:0_18:2) species following MsPAFAH as compared to HuPAFAH treatment and control in male (upper panel) and female (lower panel) worms. **(H)** MALDI-MS imaging of the MsPAFAH-hydrolyzed products of PC(16:0_20:1) and PE(18:0_22:4). Data information and statistics: Results are representative of at least three independent experiments (5 worms in each group) and are expressed as means ± SEM. For panels A, B, C, D, E, F, and G, statistical p values were calculated by ordinary one-way (A, B, G) or two-way (C, D, E, **F)** ANOVA + Dunnett´s post hoc test against HM-treated worms, *P < 0.05; **P < 0.01; ***P < 0.001; ****P < 0.0001. For panels C and D, fold changes were calculated from the relative intensities (% of total diacyl-PC or diacyl-PE) of the species from MsPAFAH- or MSe-treated against HM-treated worms. The differentially regulated species were marked when the following criteria were met: 1) the proportion of the species (% of total PC or PE) > 0.5%, 2) fold changes against HM control ≤ 0.9 or ≥ 1.1, 3) P values < 0.1 adjusted.

Next, we investigated the effects of MSe and MsPAFAH on specific membrane PLs. Interestingly, male worms responded more strongly than females (Fig 4A-4B, left panels). MSe significantly elevated total diacyl-PC levels in males, whereas MsPAFAH predominantly increased total diacyl-PE levels (Fig 4A-4B, left panels). In female worms, both MSe and MsPAFAH induced modest increases in total diacyl-PC and diacyl-PE content by trend.

Principle component analysis (PCA) of the diacyl-PC or diacyl-PE profiles revealed distinct shifts in phospholipid composition upon treatment with MSe and MsPAFAH (Fig 4A-4B lower left panels). Sex-dependent differences are primarily reflected along PC2 (y-axis) in the score plot, whereas the effects of HM versus MSe and MSe versus MsPAFAH involve contributions from both PC1 and PC2 (Fig 4A-4B lower left panels and S2D Fig). The corresponding loading plots highlight the diacyl-PC and diacyl-PE species driving these separations (Fig 4A–4B lower right panels). Species with the largest loadings along PC2, which primarily determines sex differences, include MUFA-containing phospholipids [e.g., PC(16:1_20:1), PC(18:0_18:1), PE(16:0_20:1), PE(18:0_20:1)], PUFA-containing species [e.g., PC(18:0_arachidonic acid (20:4), PE(18:0_22:4)], and lyso-PEs [e.g., 16:0-lyso-PE, 18:0-lyso-PE, 18:1-lyso-PE]. In contrast, PC1 is dominated by phospholipids containing 18:1, 18:2, 20:3, 22:5, and 22:6 [e.g., PC(18:1_18:1), PC(16:0_18:2), PC(18:0_20:3), PE(18:0_22:5), PE(18:0_22:6)).

We further explored the extent and direction in which MSe and MsPAFAH modulate the proportions of individual PL species or subgroups. Treatment with MSe or MsPAFAH substantially altered the availability of various diacyl-PC (Fig 4C) and diacyl-PE species in both male and female worms (Fig 4D). To identify diacyl-PLs consistently affected by both treatments, we displayed the data in volcano plots, highlighting PLs regulated in the same direction by MSe and MsPAFAH and thus likely representing specific MsPAFAH substrates. Additional detailed analyses are provided in S3 and S4 Figs. Differentially regulated species of interest were selected based on the following criteria: 1) relative proportion >0.5% of total diacyl-PC or diacyl-PE, 2) fold changes versus HM control ≤ 0.9 or ≥ 1.1, and 3) p value < 0.1 (MSe or MsPAFAH versus HM).

We identified a small subset of diacyl-PC or diacyl-PE species whose levels were comparably altered by both MSe or MsPAFAH in male and female worms (Fig 4C-4D). Of interest, the most abundant species, PC(16:0_20:1) and PE(18:0_22:4), were significantly decreased by both treatments regardless of sex (Fig 4E-4F). In contrast, the proportions of PC(18:0_18:2) (Fig 4E) and PE(18:0_18:2) (Fig 4F) – which account for only 1–2% of total diacyl-PC or diacyl-PE, compared with 30–50% for PC(16:0_20:1) and PE(18:0_22:4) (Fig 4E-4F) – were elevated, as confirmed by MALDI-MS imaging (Fig 4E-4F, right panels. These effects on PC and PE species were only observed with MsPAFAH, but not in the presence of HuPAFAH (Fig 4G). Interestingly, analysis of hydrolysis products of PC(16:0_20:1) and PE(18:0_22:4) by MALDI-MS imaging revealed an increase of lyso-PC(20:1) and lyso-PE(18:0) (Fig 4H), in line with the reduction of the corresponding MsPAFAH-substrate PLs.

In summary, MSe and MsPAFAH strongly impact the PL composition of *S. mansoni* by reducing abundant diacyl-PC and diacyl-PE species while increasing their hydrolysis products. Potentially, as an adaptive response to this dysregulation, the total diacyl-PL content, particularly 18:2-containing species, is substantially upregulated.

### PAFAH remodels schistosomal ether PE abundance and composition in a sex-dependent manner

**Fig 5:**
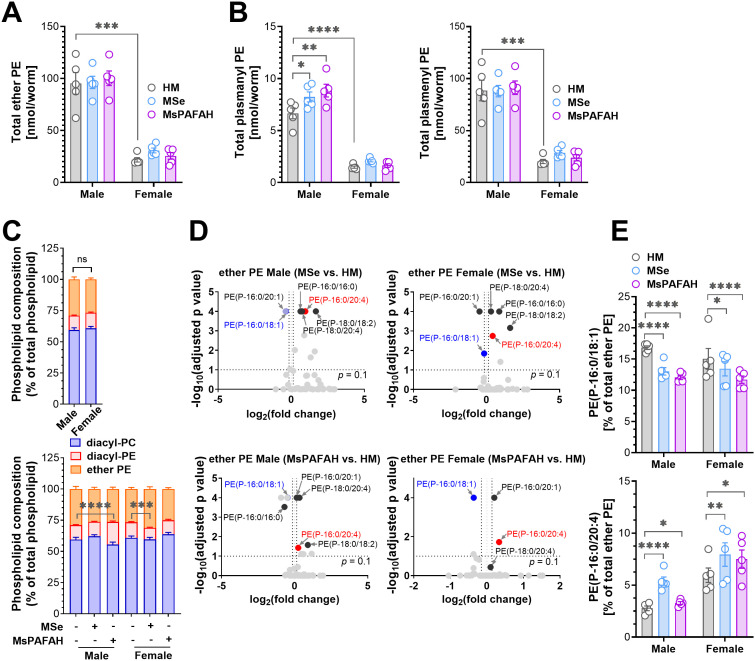
MsPAFAH remodels schistosomal ether PE abundance and composition in a sex-dependent manner. **(A)** UPLC-MS/MS analysis of the absolute amount (nmol/worm) of total ether phosphatidylethanolamine (PE) in male and female *ex-vivo* adult worms following MsPAFAH and MSe treatment. **(B)** UPLC-MS/MS analysis of the absolute amount (nmol/worm) of total plasmanyl PE (left panel) and plasmenyl PE (right panel) in male and female adult worms following MsPAFAH and Mse treatment. **(C)** Total diacyl-PC, diacyl-PE, and ether PE composition (% of total phospholipids) in male and female worms (left panel) and upon MsPAFAH and MSe treatment (right panel). **(D)** Volcano plots showing the differentially regulated ether PE species (relative intensities, % of total ether PE) upon MSe and MsPAFAH treatment in male and female worms. **(E)** Proportion of individual ether PE species (% of total ether PE). Proportions of the downregulated PE(P-16:0/18:1) (upper panel) and upregulated PE(P-16:0/20:4) (lower panel) species following MsPAFAH and MSe treatment. Data information and statistics: Results are representative of at least three independent experiments (5 worms in each group) and are expressed as means ± SEM. Statistical P values were calculated by ordinary two-way ANOVA + Dunnett´s *post hoc* test (A, B, left panel of C, D, E) or unpaired two-tailed *t*-test (right panel of **C)** *P < 0.05; **P < 0.01; ***P < 0.001; ****P < 0.0001; ns: not significant. For panel D, fold changes were calculated from the relative intensities (% of total PC or PE) of the species from MsPAFAH- or MSe-treated against HM-treated worms. The differentially regulated species were marked when the following criteria were met: 1) the proportion of the species (% of total ether PE) > 0.5%, 2) fold changes against HM control ≤ 0.9 or ≥ 1.1, 3) P values < 0.1 adjusted.

The substrate specificity of plasma PAFAH is not limited to PAF, diacyl-PC, and diacyl-PE but also includes ether- and oxidized PLs [57,61]. In fact, plasmanyl and plasmenyl PE (with an ether-bound fatty alcohol at the sn-1 position and an acyl group at the sn-2 position) constitute a substantial fraction of the *S. mansoni* tegument membrane PLs [26,50]. As with total diacyl-PE and diacyl-PC, overall baseline levels of ether PE were higher in males as compared to female worms, similarly for plasmenyl PE and plasmanyl PE (Fig 5A-5B). However, although no differences were seen for total ether PE and plasmenyl PE following exposure to MsPAFAH and MSe, we observed an increase of plasmanyl PE levels in adult male worms in contrast to females (Fig 5A-5B). Furthermore, MSe and MsPAFAH inversely altered the ratio of diacyl-PC, diacyl-PE, and ether PE (Fig 5C) in male and female worms, thereby revealing gender differences due to treatment which are not present at baseline in untreated worms (Fig 5C).

We further analyzed whether MSe and MsPAFAH affect the proportions of individual ether PE species in male and female worms. Overall, MSe was more effective than MsPAFAH in remodeling the ether PE composition. Of note, shared effects of MSe and MsPAFAH between males and females are mainly the decrease in the proportion of PE(P-16:0/18:1) and the increase in the proportion of PE(P-16:0/20:4) (Fig 5D upper and lower panels). The effects were more pronounced in males than in females, as shown by the example of PE(P-16:0/18:1) (Fig 5E, left panel) and PE(P-16:0/20:4) (Fig 5E, right panel). Importantly, the stronger effect of MSe, as compared to MsPAFAH, in modulating the proportion of these species suggests additional factors present in MSe involved in their regulation.

Together, exposure to MsPAFAH, and to a lesser extent to MSe, alters the ether PL composition, with an increase in the total plasmanyl ether PE levels, preferentially in male *S. mansoni* worms, with commonalities between males and females in some unsaturated plasmenyl PE species.

### Supplementation of fatty acids and precursors depleted from phospholipids by PAFAH exposure attenuates PAFAH’s toxicity preferentially in female worms

**Fig 6:**
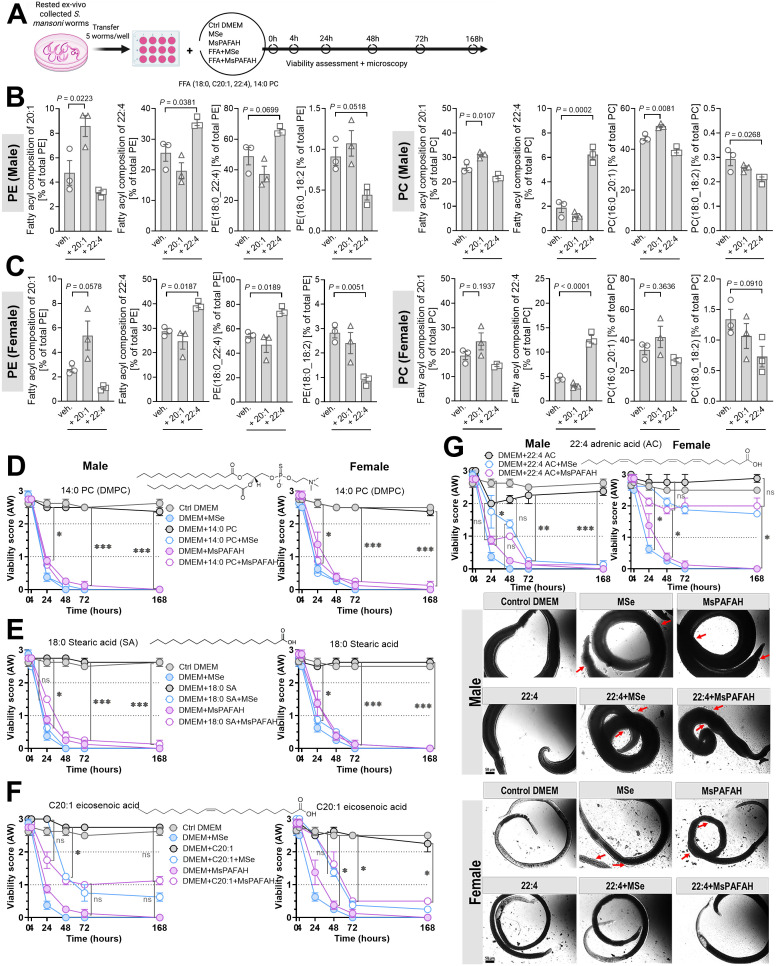
Supplementation of fatty acids and precursors depleted from phospholipids by PAFAH exposure attenuates PAFAH toxicity preferentially in female worms. **(A)** Experimental design of fatty acid supplementation and viability testing. Created in BioRender. https://BioRender.com/mecph08. (B(B) – (C) Respective diacyl-PE (left panels) and diacyl-PC (right panels) composition following fatty acid and precursors 20:1 and 22:4 supplementation to ex vivo recovered male (B) and female (C) adult worms in comparison to DMEM control (veh.) at 72 h. (D) - (G) Distinct fatty acid supplementation to *ex vivo* recovered male (left panel) and female (right panel) adult worms alone and in combination with MsPAFAH or MSe in comparison to DMEM control and viability assessment over time. A representative picture of each condition is shown in G for the supplementation of 22:4 adrenic acid. Red arrows show the effect of MsPAFAH and MSe, and in combination with 22:4 adrenic acid, on males and females. Data information and statistics: Results are representative of at least two to three independent experiments (5 worms per condition) and are expressed as means ± SEM. Asterisks show significant statistical differences analyzed using ordinary one-way ANOVA + Dunnett´s post hoc test (B - C) and two-way ANOVA followed by Tukey’s multiple comparison test (D-G). *P < 0.05; **P < 0.01; ***P < 0.001; ****P < 0.0001.

Our current data revealed that MsPAFAH induces remarkable changes in the PE and PC composition of *S. mansoni*, which correlate positively with decreased worm viability. In particular, it significantly reduced the proportion of the dominant schistosomal PL species, PC(16:0_20:1) and PE(18:0_22:4), irrespective of sex. Schistosomes have lost the ability to synthesize fatty acids *de novo*, but they can incorporate and modify fatty acids by chain elongation to build complex lipids when supplied with dietary sources [25,26]. This led us to test whether supplying exogenous FFA to MSe- and MsPAFAH-treated male and female adult worms would prevent the decline in viability and inhibit parasite death (Fig 6). To this end, we supplemented adult worms with the FFA C20:1/eicosenoic acid and 22:4/adrenic acid during treatment with MSe and MsPAFAH to compensate for the reduction in PC(16:0_20:1) and PE(18:0_22:4), respectively (Fig 6A). As expected, the supplementation of these FAs significantly increased the content of 20:1 and 22:4 in PE and PC in both male (Fig 6B) and female (Fig 6C) worms and compensate MSe- and MsPAFAH-induced disruption of these PE and PC species (S5A-S5B Fig). 18:0/stearic acid and DMPC (1,2-dimyristoyl-*sn*-glycero-phosphocholine) (major species not expected to be limiting) were supplemented as controls. Indeed, neither 18:0 stearic acid nor DMPC supplementation rescued worm viability after MSe and MsPAFAH treatment (Fig 6D-6E). However, kinetic studies revealed that supplementation with C20:1/eicosenoic acid tended to impair and delay the MSe/MsPAFAH-induced decrease in viability, especially in male worms (Fig 6F). More strikingly, supplementation with 22:4/adrenic acid greatly delayed the MSe- and MsPAFAH-induced killing in males and largely prevented the decrease of viability in females up to 168 h (Fig 6G). Of note, none of the supplemented lipids interfered with the viability of untreated worms (Fig 6D-6G).

Microscopically, male and female parasites incubated in the presence of MSe and MsPAFAH alone appeared contracted and heavily granulated with disrupted tegument after 72 h incubation (Fig 6G, middle and right panels). Additionally, the worm tegumental tissue structure was remarkably affected showing bubble formation on the tegumental surface (red arrows). Unlike in male worms, the addition of 22:4/adrenic acid into the culture system abrogated the bubble formation and granulation in female worms, rendering their appearance comparable to untreated or 22:4 only treated worms.

Our data indicate that MsPAFAH-induced toxicity in *S. mansoni* is attenuated by supplying fatty acids, i.e., 22:4/adrenic acid and to a lesser extent C20:1/eicosenoic acid, which are crucial for the biosynthesis of membrane PLs that are preferentially depleted by MsPAFAH treatment. These effects were most evident at 24–48 h and less at 72 h, except for 22:4/adrenic acid in female worms, where consistent protection against worm death was observed up to 168 h.

Together, these results provide strong evidence for the critical role of PAFAH present in mouse serum as a potent schistosomal multi-stage agent in controlling the maintenance of tissue integrity and survival of *S. mansoni* by targeting schistosome body and tegumental PE and PC, specifically 20:1- and 22:4-containing species.

## Discussion

Regulated host-parasite interaction is a prerequisite for the survival of both the host and the parasite. Parasites, in particular, rely on specific adaptations to the host and the anatomic niche in which they reside. In the case of schistosomes, it is the blood system. Therefore, understanding the underlying developmental prerequisites for survival, such as interaction with soluble serum factors, opens avenues for better understanding host-specificity. This offers promise to identify suitable targets and schistomicidal mechanisms of host factors and molecules. In the case of schistosomes, mice can be a naturally occurring reservoir host despite being widely used as an experimental, preclinical model [10,27,62]. We describe here, the identification of a soluble serum factor from mice, the platelet-activating factor acetylhydrolase (PAFAH), which is a bioactive enzyme and phospholipase present in mouse serum, as a highly potent schistosomicidal factor. We also identified other molecules (e.g., MUP10, adiponectin) which had schistosomicidal effects, however to a lesser extent and at higher concentrations, which could also contribute to the overall very strong killing effect of MSe. However, we focused in this study on deciphering the mode-of-action of PAFAH since it demonstrated the strongest schistosomicidal *in vitro* and *ex vivo* activity across all parasite developmental stages present in the definite host. We indeed found that PAFAH dysregulated specific parasite phospholipids, particularly certain species thereof (PE, PC) which overall demonstrates that the PL metabolism, which is central to maintaining tegument integrity and is thus one of the parasites’ Achilles heels, could open an avenue for pharmacological targeting in the future. While our findings underscore the significant role of MsPAFAH in regulating phospholipid remodeling/metabolism, critical for the survival of *S. mansoni*, the current study predominantly relies on *in vitro* and *ex vivo* methodologies, which, while robust and controlled, may not fully capture the complexity of host-parasite interactions in *in vivo* settings. Translating these findings into therapeutic applications warrants further investigation.

Until now, investigations into targeting the parasite lipid metabolism for drug development are scarce. While previous studies provided valuable insights into the broader role of phospholipases in helminth biology and immune evasion, they failed to address how helminths, especially schistosomes, may interact with host lipid metabolism-related enzymes, such as PAFAH, during infection [26,51,55,63,64]. Our findings reveal an increase of PAFAH, upon schistosome infection, in mouse serum, which we previously demonstrated to harbor soluble schistosomicidal factors [10], suggesting a key role of MsPAFAH in host-parasite interactions and the establishment and progression of *S. mansoni* infection in mice. In general, PAFAH is known to hydrolyze sn-2-acyl-bioactive lipids, especially the platelet-activating factor (PAF) [57,61], and has previously garnered attention for its diverse physiological functions, including the modulation of immune responses and inflammatory processes in the host [11,23,57,65–67]. Interestingly, we noted a discrepancy in the impact of the anti-schistosomal capacity of PAFAH on the development and survival of schistosomes depending on the species of origin. Indeed, while MsPAFAH had detrimental effects on the survival and development of *S. mansoni ex vivo*, HuPAFAH instead promoted parasite survival and, subsequently also, development, as demonstrated previously [10,27]. Indeed, host PAFAH activity is closely related to its interactions with soluble lipoproteins, with high-density lipoprotein (HDL) mainly favoring a higher activity than low-density lipoprotein (LDL) [57,68]. In humans, 70% of plasma PAFAH is associated with LDL and only 30% with HDL [57,68]. In contrast, up to 70% of plasma PAFAH in mice is bound to HDL and expresses higher protein activity levels than species associated with LDL and low HDL particles [57,68]. Thus, the activity and target specificity of MsPAFAH are almost nine times higher than that of the human enzyme [69,70]. Specifically, PAFAH is well known to circulate in association with serum lipoproteins, and this association differs between species. These species-specific differences occur from variations in the N-terminal region of PAFAH, which mediates specific protein-protein interactions with lipoprotein particles [71,72]. Importantly, lipoprotein binding influences the stability, localization, and catalytic efficiency of PAFAH, as well as its access to phospholipid substrates, with HDL binding being more efficient [71]. Based on these observations, our hypothesize is that the different lipoprotein associations of mouse and human PAFAH may influence the enzyme’s ability to access and hydrolyze phospholipid substrates within the schistosome tegument and membranes. HDL-associated PAFAH in mice may stabilize PAFAH binding and provide improved accessibility to parasite membrane phospholipids and oxidized lipid substrates, thereby enabling the lipid-remodeling activity that ultimately compromises parasite viability. In contrast, the LDL-associated human enzyme may have reduced access to relevant parasite phospholipid substrates under the experimental conditions we used. This may explain the delayed activity of MsPAFAH in the presence of the HuPAFAH we observed.

In addition, the comparison of the HuPAFAH structure with the AlphaFold2-derived model of MsPAFAH (sequence alignment) clearly showed that while the catalytic triad is perfectly conserved across the 5 mammalian species and the HDL binding site is very similar in both mouse and human orthologs, there are marked differences in the LDL binding site. Specifically, the triad Trp115/Leu116/Tyr205 is involved in LDL binding in HuPAFAH, but only Tyr205 is present in MsPAFAH, potentially explaining why MsPAFAH is mainly associated with HDL in mice, while HuPAFAH is mainly associated with LDL. This is in all likelihood an adaptation to the fact that rodents such as mice, rats, and guinea pigs express little LDL due to a lack of cholesteryl ester transfer protein [73]. Comparing the AlphaFold2 predicted structures for human and mouse PAFAH, the root-mean-square deviation (RMSD) between mouse and human PAFAH over 361 pruned atoms is 0.569 Å. The RMSD between the solved human PAFAH structure (3D59) over 363 pruned atom pairs is 0.636 Å. These exceptionally low values indicate near identity of the two proteins in the vicinity of the active site and across most of the molecules, suggesting that the difference in schistosomicidal activity is unlikely to arise from catalytic center alterations, but rather in regions implicated in lipoprotein association and substrate accessibility.

Nevertheless, although the schistomicidal effects of MSe and MsPAFAH were similar in most cases, we sometimes observed a more potent killing effect of MSe, suggesting the potential presence of additional factors in serum with added effects, such as in F1 fraction, in which

additional active components need to be investigated and the mechanistic pathways addressed. As discussed above, notably, mouse PAFAH is more likely to be predominantly associated with HDL, which supports binding stability, substrate accessibility and higher catalytic activity. In contrast, the recombinant MsPAFAH used in our assays was purified and tested as active soluble protein outside the natural lipoprotein context. The obvious absence of lipoprotein binding partners in our *in vitro* setting likely affects the access to phospholipid substrates and may therefore explain the somewhat reduced catalytic activity compared with endogenous PAFAH. Furthermore, while the precise lipid composition of the HybridoMed (HM) culture medium is not fully known, it contains lipids, cholesterol, and proteins that could potentially support lipoprotein-associated enzymatic activity of MsPAFAH in *in vitro* short-term culture with no host serum supplementation. Also, *in vivo*, there might be endogenous inhibitors of PAFAH that are not present in the *in vitro* assay. Thus, eventually, the effects of MSe and MsPAFAH killing *in vitro* might be distinct in *in vivo* settings and need further investigation. In our view, the presence of anti-schistosomal activity in MSe and the survival of a subset (e.g., 30% after infection) of worms in mice are not mutually exclusive. Rather, they suggest that MSe represents one component of a multifactorial host environment that restricts parasite development, especially during early migration. Parasites that successfully mature *in vivo* have likely undergone substantial physiological adaptation to the murine host. This interpretation is supported by transcriptomic studies showing that schistosomes alter gene expression during intra-mammalian migration, including upregulation of pathways involved in tegument remodeling, developmental control, gut activation, signalling, and adaptation to host-derived stress, particularly at the lung stage [74–76]. An intriguing observation in our study is that adult worms recovered from mice, despite having survived prolonged *in vivo* exposure to mouse serum, rapidly lose viability when re-exposed to MSe under *ex vivo* conditions. We speculate that this may reflect the loss of protective or compensatory mechanisms that are maintained *in vivo* by the host tissue environment and by the continuous availability of host-derived nutrients and lipids including free fatty acids, which could counterbalance the lipid-modifying activity of host anti-parasitic molecule such as PAFAH. In this context, our rescue experiments are informative, as supplementation with specific fatty acids restored parasite viability, supporting the idea that host lipid availability may buffer or compensate for PAFAH-mediated phospholipid remodeling *in vivo*, whereas this buffering capacity is reduced in simplified *ex vivo* culture conditions.

PAFAH-mediated damage was evident in the tegument and the schistosomes’ gut and reproductive organs, as revealed by CLSM and SEM, leading to an additional gross reduction in parasite reproductivity. Similar post-treatment phenotypes were previously reported for arylmethylamino steroids, a novel compound class affecting worm fitness, reproduction, and tissue morphology, including the gonads, tegument and gut [77]. Although this compound’s target(s) are unknown, tegumental membrane destabilizing effects were discussed. Previous studies have shown that the parasite tegument serves not only as a protective interface with the host environment but also as a dynamic metabolic structure involved in nutrient uptake and signaling within schistosomes [26,59]. Disruption of tegumental membrane integrity can therefore propagate systemic physiological signaling within the parasite. In this context, the structural alterations observed in the gut and reproductive organs following MsPAFAH treatment are consistent with the global disturbance of parasite phospholipid homeostasis demonstrated in our lipidomic analyses. The reproductive organs of schistosomes are particularly sensitive to membrane perturbations, as germ cell proliferation, differentiation, and pairing stability depend on tightly regulated cellular signaling and membrane dynamics [78,79]. Similarly, the schistosome gut epithelium we analysed is highly membrane-rich and essential for nutrient acquisition and metabolic exchange. Our imaging analyses show that MsPAFAH treatment is associated with disorganization of reproductive tissues and impaired gonadal stem cell proliferation and differentiation. This suggests that the disruption of membrane phospholipids may generate bioactive lipid mediators which interfere with cellular signaling and tissue organization in these organs, affecting cellular homeostatis leading to parasite death. Membrane and tegument phospholipids as potential PAFAH targets are essential for schistosome survival and are increasingly recognized as mediators, or precursors thereof, in signal transduction and immune response modulation [26,50]. Thus, tegument-specific phospholipids play a substantial role in host-parasite interactions, as evidenced by the roles of lyso-PC and lyso-phosphatidylserine in schistosomal host immune evasion [80,81]. These are also crucial for various pathophysiological processes related to cell survival and death of eukaryotes in general; for example, 1,2-dioleoyl-*sn*-glycero-phosphoinositol (PI(18:1/18:1)) in stress adaption [60], oxidized phospholipids in apoptosis and ferroptosis [82], and lipid mediators in inflammation [83], among others. We thus investigated whether PAFAH targeted specific phospholipid species for hydrolysis, disrupting tegumental membrane integrity and allowing the influx of damaging ions and other molecules, eventually activating further internal signaling.

Notably, treatment with MsPAFAH, similarly to MSe, elevated total PC and PE levels, especially in male *S. mansoni*. This suggests a compensation mechanism in response to the MsPAFAH-mediated hydrolysis of lipids, which might correlate to schistosomes’ reported rapid membrane renewal capabilities [49]. This renewal of the membrane complex is closely associated with increased schistosome lipid metabolism and related enzymes following stress events [84]. These enzymes (e.g., acyltransferases present in schistosome) may be used by the worms to incorporate MsPAFAH-hydrolysis products (e.g., FFA and lyso-PL) into various phospholipids species [85], such as PC and PE, thus compensating for membrane PL loss following MsPAFAH-dependent degradation, an effect we observed when we supplemented FFA to MSe or MsPAFAH treated schistosomes. Other studies suggested that the tegument is constantly renewed, either by acylation and deacylation of specific lipids directly in the tegument, and that the male worms shed their outer layer of the tegument, enriched in phospholipids, to potentially get rid of antibodies and attacking immune cells, resulting yet again in the upregulation of phospholipid synthesis and levels [15,49], especially in male worms. In line with our observations, previous electron microscopic investigations have identified LDL, but also HDL, to be attached to their receptors, the glycosylphosphatidylinositol (GPI)-linked low-molecular-weight proteins, on the outer layer of the tegument and dorsal regions of *ex vivo* flushed adult worms [86]. Thus, HDL/LDL-bound PAFAH would potentially attach and hydrolyze tegument phospholipids, producing *S. mansoni* specific fatty acids (FA) and/or LysoPLs. In addition, bound HDL and LDL activate the cytons to produce membranous vacuoles to replace the missing tegument. The continuous tegumental shedding may disrupt the inner tegument layer and eventually lead to parasite death, especially in male schistosomes, possibly further explained by the fact that male schistosomes preferentially attach LDL and HDL particles to their tegument [86]. Indeed, as previously reported, such sex-dependent differences with more effect on male worms were also observed along with higher PL content in males compared to females [50,86]. Whether the higher susceptibility of male worms to treatment is functionally linked to the higher content of total PLs or related to differences in the FA composition currently remains elusive.

Importantly, specific PLs, including ether PLs containing FA characteristic for schistosomes (e.g., 20:1, 18:0, 18:1(5Z)), are enriched in the tegument and have been shown to determine the parasite susceptibility to stress and death signals [15,49]. These lipids may serve as feedback signals to underlying tegumental cells, reflecting tegument integrity [15,49]. To identify parasite-specific tegumental PLs, whose abundance is modulated by MsPAFAH, we performed a comprehensive quantitative metabololipidomic analysis focusing on affected PC and PE species. Given the complexity of lipid metabolism and the limited functional annotation of *S. mansoni* orthologs, it remains challenging to assign the observed changes to specific sex-dependently regulated metabolic pathways. Comprehensive multiomics studies linking lipidomic alterations to transcriptional and translational regulation of lipid metabolic enzymes, together with functional studies involving gene manipulation, are currently lacking and are beyond the scope of the present study, representing an important limitation. Direct comparisons with other eukaryotes are further complicated by the atypical and only partially understood lipid metabolism of *Schistosoma* species. Notably, schistosomes are unable to synthesize fatty acids *de novo* and instead rely on host-derived precursors for the biosynthesis of complex lipids such as PC and PE [25]. Fatty acids are transported intracellularly by fatty acid-binding proteins, activated as CoA esters, and can undergo elongation, including the dynamically favored conversion of 18:1-CoA to 20:1-CoA [25,87,88]. This pathway may also support the formation of PUFA species such as 22:4/adrenic acid from precursors like 20:4/arachidonic acid. Several candidate elongases have been annotated based on sequence similarity but remain functionally uncharacterized (Smp_010770.2, Smp_051810, Smp_130370, Smp_010720) [87]. To date, potential sex-dependent regulation of elongases or other fatty acid–metabolizing factors has not been investigated.

Our metabolipidomics analyses revealed substantial changes in the phospholipids profile (> 100 species), most prominently affecting the abundant species PC(16:0_20:1) and PE (18:0_22:4), whose proportions were reduced in both male and female worms following MsPAFAH treatment and, as such, may potentially represent specific (direct or indirect) targets of MsPAFAH. Interestingly, both MsPAFAH and MSe increased the proportion of PC(18:0_18:2) and PE(18:0_18:2) along with other diacyl- and ether-PC and -PE species in worms of both sexes. These pronounced and coordinated changes imply the activation of several complex defense mechanisms in schistosomes by MsPAFAH treatment, damaging the worms’ tegument, gut, and reproductive organs. Remarkably, supplementation with the respective FFAs depleted from membrane PLs upon MsPAFAH treatment almost entirely prevented the MsPAFAH-induced decrease in female viability, while protection was only partial in males. This sex-specific effect is an essential finding with potential *in vivo* relevance concerning schistosome fecundity, reproductivity, and associated pathology. Consistently, paired female schistosomes have been demonstrated to rapidly incorporate, oxidize, modify, and turn over FAs, processes that are essential for egg production and integrity [89,90].

The mechanistic basis for the sex-dependent susceptibility to PAFAH remains incompletely defined but may arise from differences in tegument PL composition between sexes. Indeed, the female tegument contains a higher proportion of PLs, including PC and PE, than the male tegument, as previously reported [90] and confirmed here. Notably, females exhibit elevated levels of 22:4-containing PL species, whereas 20:1-containing species are relatively depleted compared with males. Because PAFAH reduces the abundance of both 20:1- and 22:4-containing PLs, it is tempting to speculate that co-supplementation with these fatty acids affords more effective protection in the sex with a higher basal requirement for – and apparently greater metabolic capacity to incorporate – the respective fatty acid into tegument PLs. Consistent with this hypothesis, supplementation with 22:4 protected females more efficiently from PAFAH-induced toxicity than males, whereas the preferential protective effect of 20:1 supplementation in males was less pronounced.

A critical question remaining is the MsPAFAH contribution to parasite clearance or developmental attrition *in vivo* in a mouse model. In this regard, a direct *in vivo* validation would represent an important future step to determine the precise contribution of PAFAH to parasite clearance or developmental restriction in the mammalian host. However, at present, such experiments are technically challenging for several reasons (e.g., a global PAFAH-knockout mouse model is currently not available). Importantly, this study identifies a host-derived enzymatic mechanism capable of disrupting schistosome phospholipid metabolism, which provides a conceptual framework for future investigations into host determinants of parasite permissiveness. Thus, at this point, altogether, rather than proposing PAFAH itself as a biological contributing to parasite clearance *in vivo*, our findings highlight parasite phospholipid metabolism as a vulnerable axis that may guide the identification, repurposing and development of anti-schistosome small-molecules inspired by the enzymatic mechanism we described in our study. Such compounds could indeed interfere with the parasite’s phospholipid metabolism or alter the fatty acid composition of tegument membranes, thereby reproducing key aspects of the PAFAH-induced phenotype described in our study. This strategy could open new avenues for anti-schistosomal molecule discovery, particularly in the context of the limited number of currently available therapeutic options and the reliance on praziquantel as the main treatment. Finally, the observed functional divergence between mouse and human PAFAH also highlights how host lipid metabolism and lipoprotein-associated enzymes may contribute to host-specific permissiveness to schistosome infection. Understanding these differences may provide valuable insight into the constraints that shape parasite survival in different host species and may help identify targeted metabolic pathways in human schistosomiasis.

## Conclusions

We provide here new insight into the critical and under researched area of parasite-host specificity, deepening our understanding of the underlying factors of host-parasite interaction during schistosomiasis. To capitalize on this discovery for developing novel therapeutics against schistosomiasis, future studies should prioritize evaluating the efficacy of MsPAFAH or ideally analogues and small molecules thereof *in vivo* models that mimic the dynamic biological environment of mammalian hosts. Further characterization of the functional PAFAH-induced changes in the lipid composition would unravel the mechanisms that control candidate bioactive lipids. This will allow us to understand the mode of action of PAFAH in more detail, address fundamental aspects of schistosome biology, reproductive development, and survival, and potentially identify accessible and druggable targets.

## Supporting information

S1 Fig Large-scale fractionation of mouse serum using Supradex 200 size exclusion column and candidate molecule identification. (A) Mouse serum fractionation and pooling of recovered fractions (F1-5). (B) Left panel: heating of mouse serum active fraction F2 at different time points and effect on NTS viability at day 3. Middle panel: Pronase E (PrE)-digested mouse serum fraction F2 and PBS (as control) and effect on NTS viability at day 3. Right panel: Proteinase K treatment of mouse serum F2 fraction and effect on NTS viability at day 3. (C) Venn diagram of refined proteins in active and inactive mouse serum fractions and heated fraction F2 with the candidate molecules. (D) Concentration-dependent effect of MsPAFAH as compared to MSe and HM control on NTS at day 3. (E) Enzymatic activity of recombinant MsPAFAH (44 µg/ml) as compared to infected (Inf) and non-infected (Ninf) MSe, and to positive control (HuPAFAH) and PBS. (F) Concentration-dependent effect MUP10, Adiponectin, and FAP as compared to Mse and HM control on NTS at day 3. (G) Effect of MSe and MsPAFAH on NTS viability in the presence or absence of the PAFAH selective inhibitor Darapladib (DAR) at day 3. (H) Left panel: comparative timeline of active (MsPAFAH)- and inactive (inMsPAFAH) PAFAH-killing efficiency. Right panel: enzymatic activity of active and inactive PAFAH.
(TIF)

S2 Fig Sex differences of tegument diacyl-PC and -PE profiles in *S. mansoni* adult worms. Lipids were extracted from male and female S. mansoni worms, phosphatidylcholine (PC) and phosphatidylethanolamine (PE) were analyzed by UPLC-MS/MS. (A) Total diacyl-PC and -PE amount (pmol/worm). (B) The proportion of individual diacyl-PC,- PE species and fatty acid distribution (% of total diacyl-PC or -PE; SFA: saturated fatty acids, MUFA: monounsaturated fatty acids, PUFA: polyunsaturated fatty acids). The color code represents the fold change (% of the mean of male worms). The numbers indicate the mean values of the individual groups, and the differentially regulated species with P < 0.1 were marked by squares. (C) The proportion of the exemplary PC(16:0_20:1) and PE(18:0_22:4). (D) Left panel: PCA score clustering sex-dependent effect of MsPAFAH and MSe treatment on total diacyl-PC. PCA plots were generated using the relative intensities of individual diacyl-PC species (% of total diacyl-PC). Right panel: PCA score clustering sex-dependent effect of MsPAFAH and MSe treatment on total diacyl-PE. PCA plots were generated using the relative intensities of individual diacyl-PE species (% of total diacyl-PE). Data are presented as A, C) mean ± S.E.M. or B) mean, n = 5 worms for each sex. *P < 0.05, ****P < 0.0001, two-tailed unpaired student t-test.
(TIF)

S3 Fig Diacyl-PC profile of the *S. mansoni* adult worms after MSe and MsPAFAH treatment. Lipids were extracted, phosphatidylcholine (PC) was analyzed by UPLC-MS/MS. The proportion of individual diacyl-PC (% of total diacyl-PC) and fatty acid distribution (% of diacyl-total PC; SFA: saturated fatty acids, MUFA: monounsaturated fatty acids, PUFA: polyunsaturated fatty acids). The color code represents the fold change (% of the mean of HM control). The numbers indicate the mean values of the individual groups and the differentially regulated species. Statistical P values were calculated by ordinary two-way ANOVA + Dunnett´s post hoc tests with P < 0.1 were marked.
(TIF)

S4 Fig Diacyl-PE profile of the *S. mansoni* adult worms after MSe and MsPAFAH treatment. Lipids were extracted, phosphatidylethanolamine (PE) was analyzed by UPLC-MS/MS. The proportion of individual diacyl-PE (% of total diacyl-PE) and fatty acid distribution (% of total diacyl-PE; SFA: saturated fatty acids, MUFA: monounsaturated fatty acids, PUFA: polyunsaturated fatty acids). The color code represents the fold change (% of the mean of HM control). The numbers indicate the mean values of the individual groups and the differentially regulated species. Statistical P values were calculated by ordinary two-way ANOVA + Dunnett´s post hoc test with P < 0.1 were marked.
(TIF)

S5 Fig Supplementation of fatty acids and precursors increases the uptake of respective PE and PC species by male worms. (A-B) Respective diacyl-PE (upper panels) and diacyl-PC (lower panels) composition following fatty acid and precursors supplementation to *ex vivo* recovered male (A) and female (B) adult worms in combination with MsPAFAH or Mse in comparison to DMEM control (veh.) Results are representative of at least two-three independent experiments (5 worms per condition) and are expressed as means ± SEM. Asterisks show significant statistical differences analyzed using ordinary One-Way ANOVA + Dunnett´s post hoc test. *P < 0.05; **P < 0.01; ***P < 0.001; ****P < 0.0001.
(TIF)
