# Supplementary figures and images for: Host-specific platelet-activating factor acetylhydrolase selectively remodels diacylglycerophospholipids to control schistosome development
Source: PLoS Pathog. 2026 May 12;22(5):e1014207. doi: 10.1371/journal.ppat.1014207 (PMC13186347; doi:10.1371/journal.ppat.1014207)

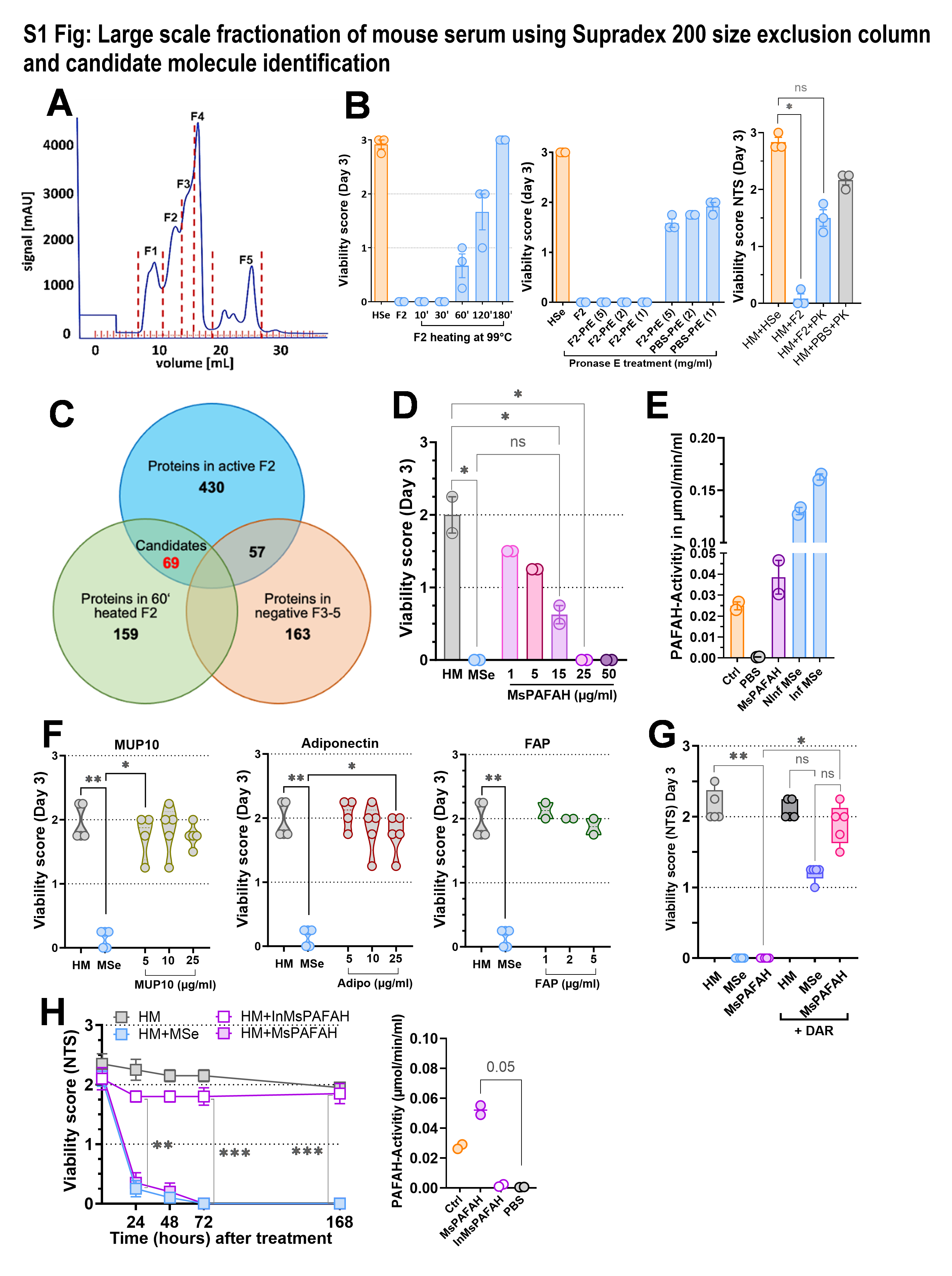

Supplement: S1 Fig — (A) Mouse serum fractionation and pooling of recovered fractions (F1-5). (B) Left panel: heating of mouse serum active fraction F2 at different time points and effect on NTS viability at day 3. Middle panel: Pronase E (PrE)-digested mouse serum fraction F2 and PBS (as control) and effect on NTS viability at day 3. Right panel: Proteinase K treatment of mouse serum F2 fraction and effect on NTS viability at day 3. (C) Venn diagram of refined proteins in active and inactive mouse serum fractions and heated fraction F2 with the candidate molecules. (D) Concentration-dependent effect of MsPAFAH as compared to MSe and HM control on NTS at day 3. (E) Enzymatic activity of recombinant MsPAFAH (44 µg/ml) as compared to infected (Inf) and non-infected (Ninf) MSe, and to positive control (HuPAFAH) and PBS. (F) Concentration-dependent effect MUP10, Adiponectin, and FAP as compared to Mse and HM control on NTS at day 3. (G) Effect of MSe and MsPAFAH on NTS viability in the presence or absence of the PAFAH selective inhibitor Darapladib (DAR) at day 3. (H) Left panel: comparative timeline of active (MsPAFAH)- and inactive (inMsPAFAH) PAFAH-killing efficiency. Right panel: enzymatic activity of active and inactive PAFAH. (TIF) [file ppat.1014207.s001.tif]

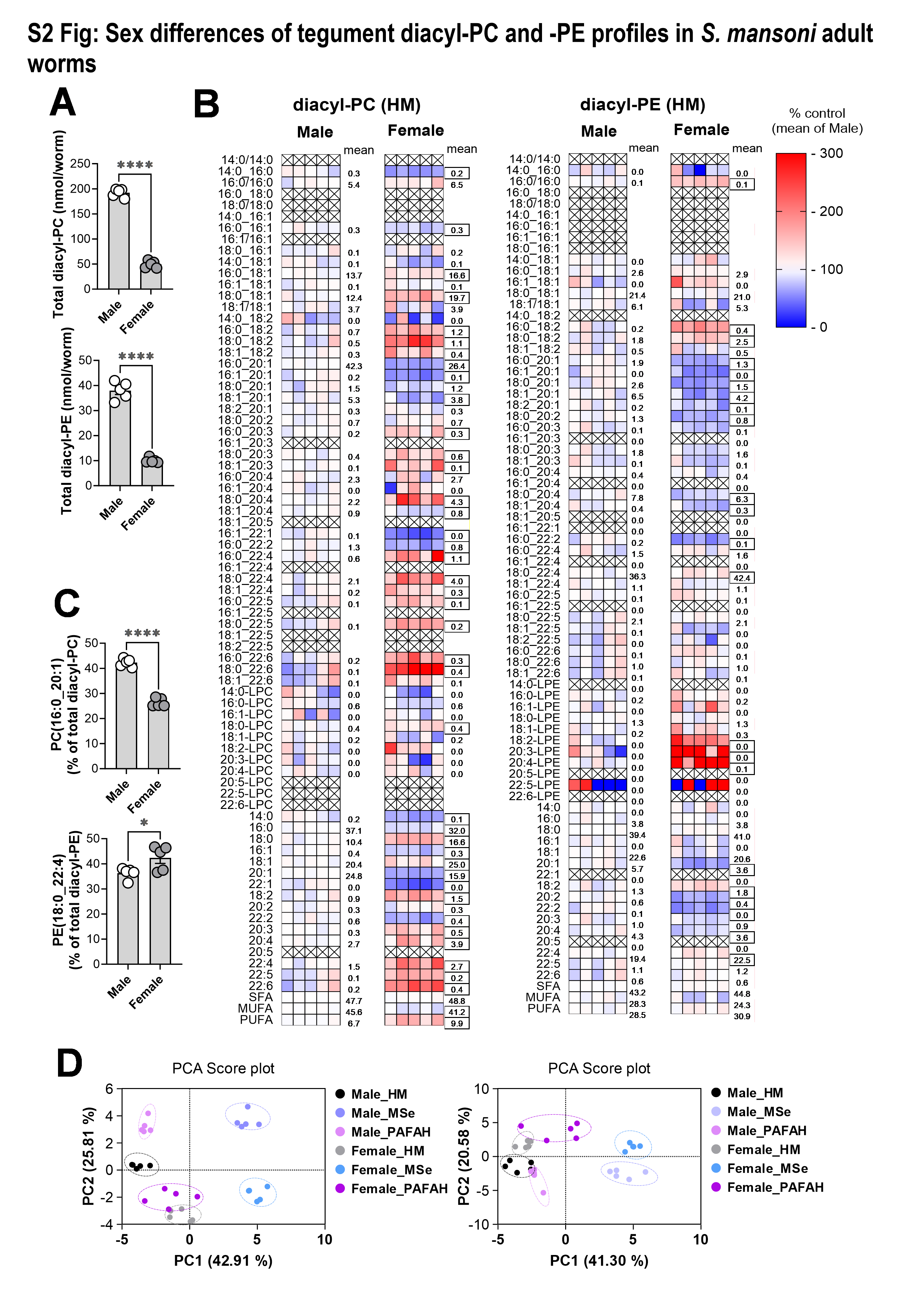

Supplement: S2 Fig — Lipids were extracted from male and female S. mansoni worms, phosphatidylcholine (PC) and phosphatidylethanolamine (PE) were analyzed by UPLC-MS/MS. (A) Total diacyl-PC and -PE amount (pmol/worm). (B) The proportion of individual diacyl-PC,- PE species and fatty acid distribution (% of total diacyl-PC or -PE; SFA: saturated fatty acids, MUFA: monounsaturated fatty acids, PUFA: polyunsaturated fatty acids). The color code represents the fold change (% of the mean of male worms). The numbers indicate the mean values of the individual groups, and the differentially regulated species with P < 0.1 were marked by squares. (C) The proportion of the exemplary PC(16:0_20:1) and PE(18:0_22:4). (D) Left panel: PCA score clustering sex-dependent effect of MsPAFAH and MSe treatment on total diacyl-PC. PCA plots were generated using the relative intensities of individual diacyl-PC species (% of total diacyl-PC). Right panel: PCA score clustering sex-dependent effect of MsPAFAH and MSe treatment on total diacyl-PE. PCA plots were generated using the relative intensities of individual diacyl-PE species (% of total diacyl-PE). Data are presented as A, C) mean ± S.E.M. or B) mean, n = 5 worms for each sex. *P < 0.05, ****P < 0.0001, two-tailed unpaired student t-test. (TIF) [file ppat.1014207.s002.tif]

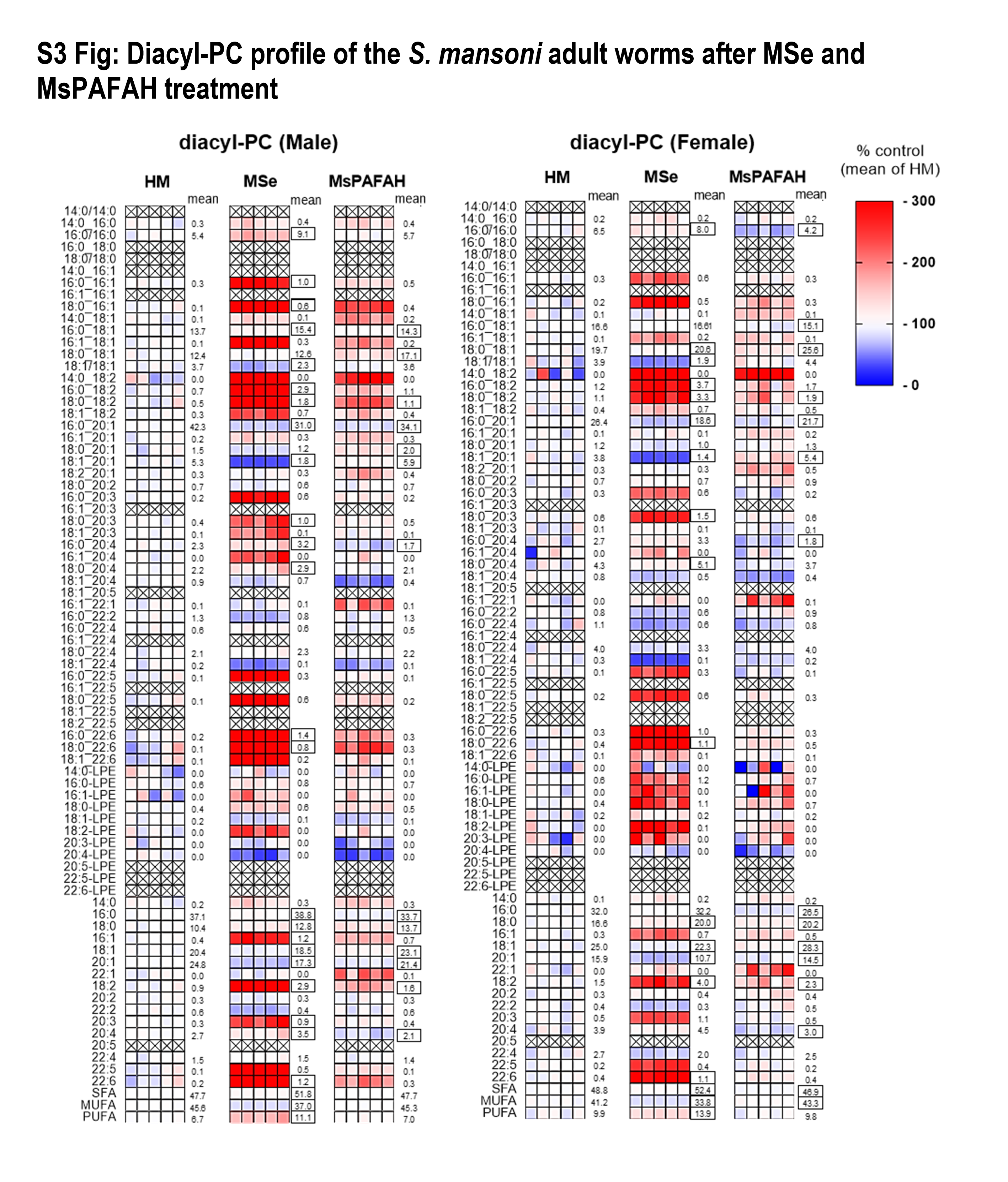

Supplement: S3 Fig — Lipids were extracted, phosphatidylcholine (PC) was analyzed by UPLC-MS/MS. The proportion of individual diacyl-PC (% of total diacyl-PC) and fatty acid distribution (% of diacyl-total PC; SFA: saturated fatty acids, MUFA: monounsaturated fatty acids, PUFA: polyunsaturated fatty acids). The color code represents the fold change (% of the mean of HM control). The numbers indicate the mean values of the individual groups and the differentially regulated species. Statistical P values were calculated by ordinary two-way ANOVA + Dunnett´s post hoc tests with P < 0.1 were marked. (TIF) [file ppat.1014207.s003.tif]

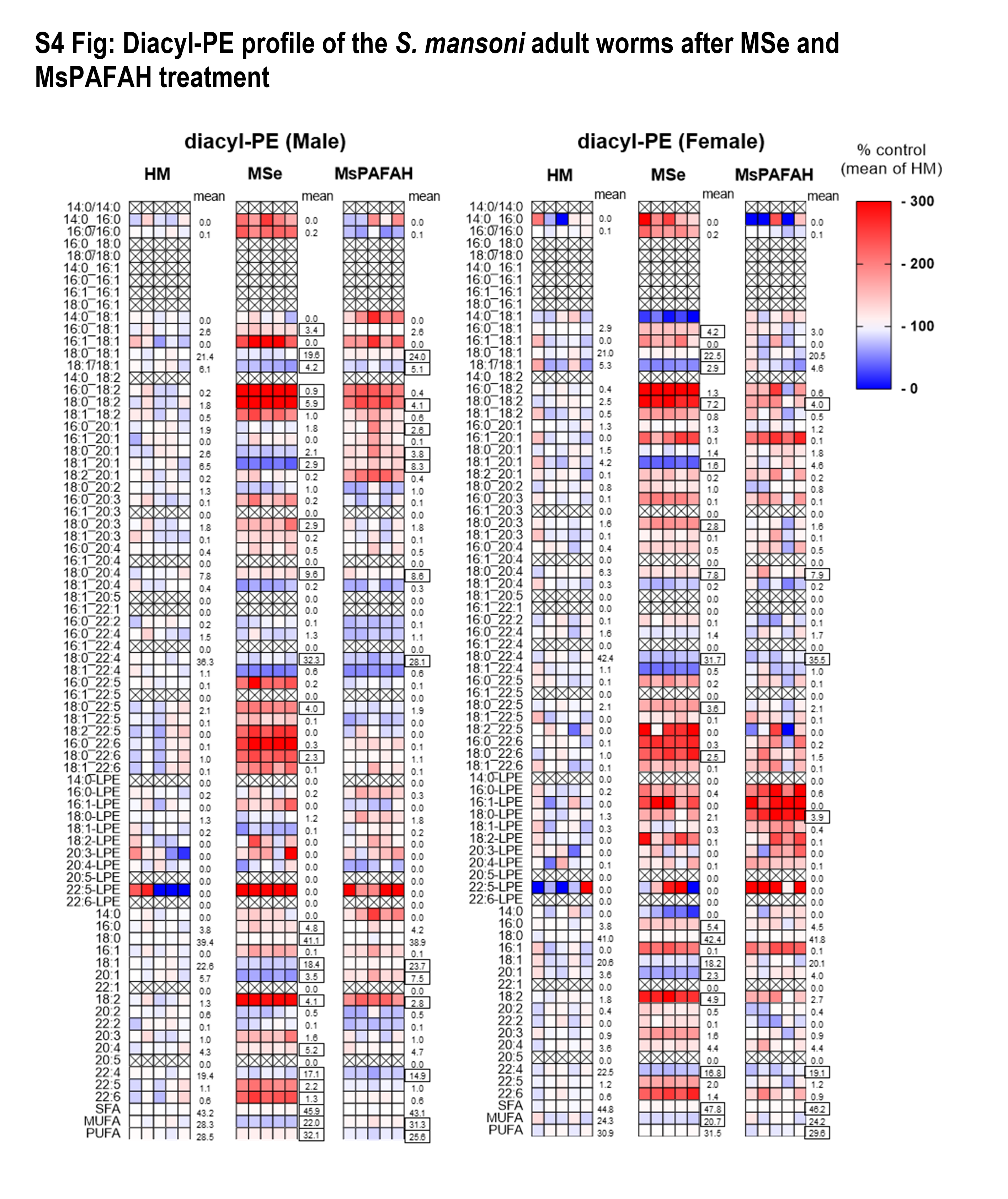

Supplement: S4 Fig — Lipids were extracted, phosphatidylethanolamine (PE) was analyzed by UPLC-MS/MS. The proportion of individual diacyl-PE (% of total diacyl-PE) and fatty acid distribution (% of total diacyl-PE; SFA: saturated fatty acids, MUFA: monounsaturated fatty acids, PUFA: polyunsaturated fatty acids). The color code represents the fold change (% of the mean of HM control). The numbers indicate the mean values of the individual groups and the differentially regulated species. Statistical P values were calculated by ordinary two-way ANOVA + Dunnett´s post hoc test with P < 0.1 were marked. (TIF) [file ppat.1014207.s004.tif]

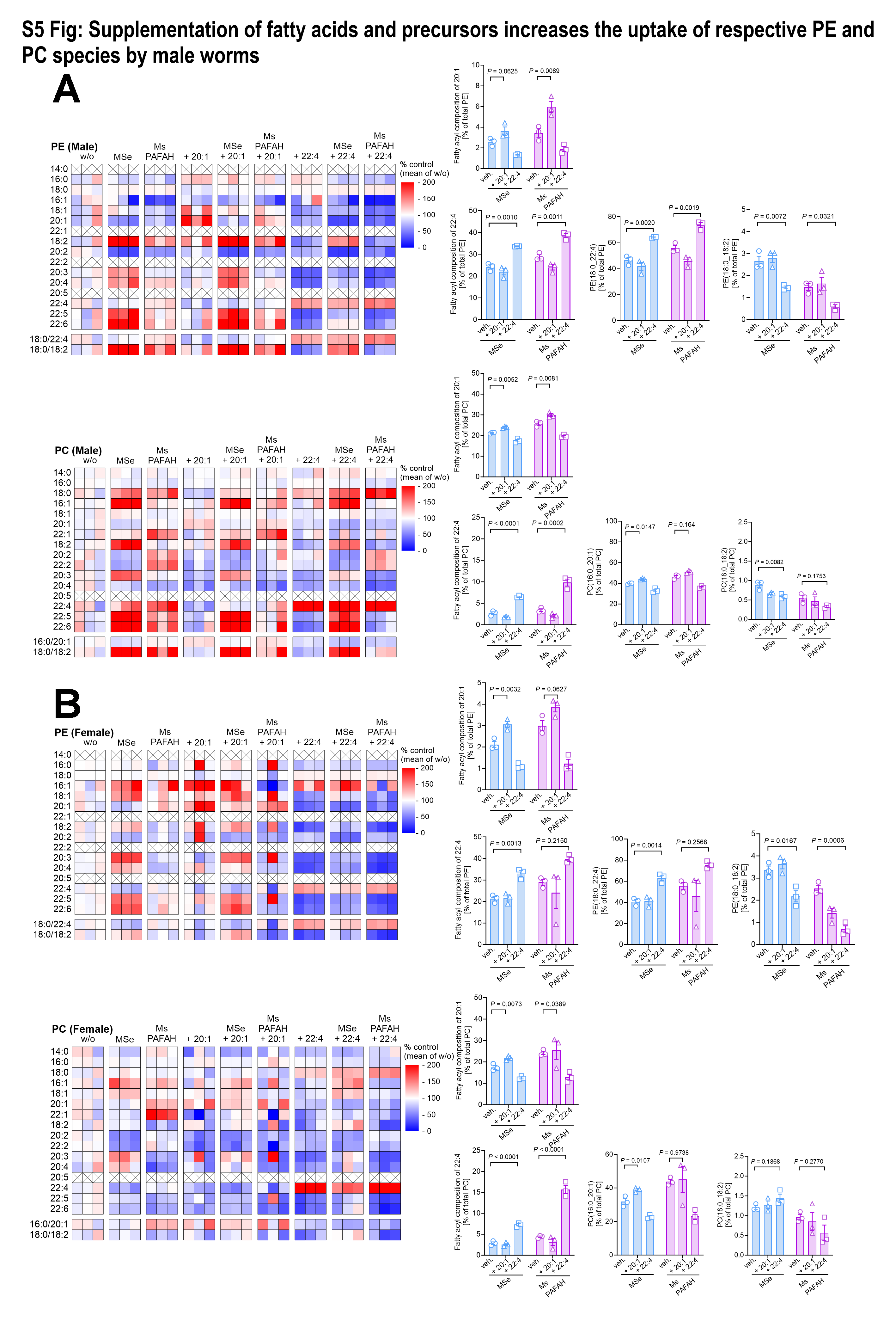

Supplement: S5 Fig — (A-B) Respective diacyl-PE (upper panels) and diacyl-PC (lower panels) composition following fatty acid and precursors supplementation to ex vivo recovered male (A) and female (B) adult worms in combination with MsPAFAH or Mse in comparison to DMEM control (veh.) Results are representative of at least two-three independent experiments (5 worms per condition) and are expressed as means ± SEM. Asterisks show significant statistical differences analyzed using ordinary One-Way ANOVA + Dunnett´s post hoc test. *P < 0.05; **P < 0.01; ***P < 0.001; ****P < 0.0001. (TIF) [file ppat.1014207.s005.tif]
